# Immediate ecotoxicological effects of short-lived oil spills on marine biota

**DOI:** 10.1038/ncomms11206

**Published:** 2016-04-04

**Authors:** Corina P. D. Brussaard, Louis Peperzak, Siham Beggah, Lukas Y. Wick, Birgit Wuerz, Jan Weber, J. Samuel Arey, Bart van der Burg, Arjen Jonas, Johannes Huisman, Jan Roelof van der Meer

**Affiliations:** 1 NIOZ Royal Netherlands Institute for Sea Research, Department of Marine Microbiology and Biogeochemistry and Utrecht University, PO Box 59, 1790 AB Den Burg, Texel, The Netherlands; 2 Department of Aquatic Microbiology, Institute for Biodiversity and Ecosystem Dynamics (IBED), University of Amsterdam, PO Box 94248, 1090 GE Amsterdam, The Netherlands; 3 Department of Fundamental Microbiology, Bâtiment Biophore, Quartier UNIL-Sorge, University of Lausanne, CH-1015 Lausanne, Switzerland; 4 Department of Environmental Microbiology, Helmholtz Centre for Environmental Research—UFZ, Permoserstraße 15, D-04318 Leipzig, Germany; 5 Environmental Chemistry Modeling Laboratory, Ecole Polytechnique Fédérale de Lausanne, CH-1015 Lausanne, Switzerland; 6 Eawag, Swiss Federal Institute of Aquatic Science and Technology, CH-8600 Dübendorf, Switzerland; 7 BioDetection Systems BV, Science Park 406, 1098 XH Amsterdam, The Netherlands; 8 Rijkswaterstaat Zee en Delta, Ministerie van Infrastructuur en Milieu, Lange Kleiweg 34, 2288 GK Rijswijk, The Netherlands

## Abstract

Marine environments are frequently exposed to oil spills as a result of transportation, oil drilling or fuel usage. Whereas large oil spills and their effects have been widely documented, more common and recurrent small spills typically escape attention. To fill this important gap in the assessment of oil-spill effects, we performed two independent supervised full sea releases of 5 m^3^ of crude oil, complemented by on-board mesocosm studies and sampling of accidentally encountered slicks. Using rapid on-board biological assays, we detect high bioavailability and toxicity of dissolved and dispersed oil within 24 h after the spills, occurring fairly deep (8 m) below the slicks. Selective decline of marine plankton is observed, equally relevant for early stages of larger spills. Our results demonstrate that, contrary to common thinking, even small spills have immediate adverse biological effects and their recurrent nature is likely to affect marine ecosystem functioning.

## Abstract (web summary)

Large oil spills have negative effects on marine ecosystems, but the effects of chronic smallscale spills are less well understood. Here, Brussaard *et al*. detect high bioavailability and toxicity of small-scale crude oil spills within 24 hours after release, which are associated with localised declines in marine plankton.

## Graphical abstract

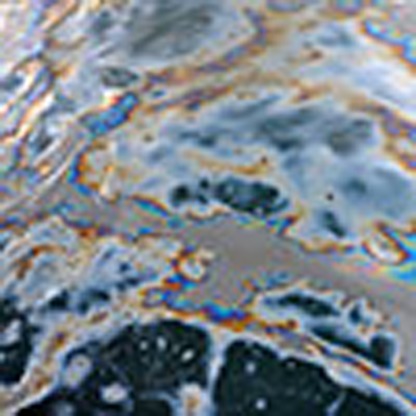

Despite important technical improvements in the safety of extraction and transport of crude oil and gas, marine environments continue to be threatened by oil spills, which can cause decades-long havoc on marine and coastal ecosystems^1^,^2^. On average, 10,000 billion tonne miles of crude oil are transported annually over sea, and an estimated 5,000 ton per year were spilled during the period 2010–2014 as a result of accidents, cleaning operations or other causes^3^. Catastrophic large spills, such as the recent Deepwater Horizon blowout in 2010 and the groundings of the Exxon Valdez in 1989 and Prestige in 2002, have released enormous quantities of oil over large areas, with disastrous immediate and long-term consequences for wildlife, coastal zones, natural resources, aquaculture activities, fisheries and/or tourism^4^,^5^,^6^,^7^. Because of improved technical standards, the number of large spills (7–700 and >700 ton) has decreased drastically during the past decades to an average of 5.2 and 1.8 per year, respectively^3^. Less well known are spills that comprise <7 ton and yet represent an estimated 80% (by number) of all recorded spills: many of such smaller spills may go unnoticed and remain unreported^3^.

The chemical changes and weathering of oil spills on sea have been well documented, and a variety of models have been developed to predict oil distribution, dissolution and weathering over time^8^,^9^,^10^,^11^,^12^. Ecotoxicological effects of (larger) oil spills have also been described in great detail, but as a consequence of the accidental nature of spills and the typical delay in detection, their early impacts are poorly documented. Dissolved and dispersed hydrocarbon concentrations in the seawater to which marine (micro)organisms are exposed will be highest during the initial stages of spills (first days)^12^,^13^,^14^,^15^,^16^. The immediate exposure is thus the major toxicity mechanism for smaller spills, which disappear (from sight) within a few days from the sea surface, although this does not necessarily mean that the oil is degraded^17^,^18^. Until now, most of the information on acute toxicity comes from the laboratory studies that have frequently used so-called water-accommodated fractions of oil^19^,^20^,^21^,^22^,^23^. In contrast, detailed studies are lacking that have simultaneously monitored dissolved oil concentrations and biological effects immediately after spills. Improved assessment of early effects, however, is a vital step towards better understanding of biological impacts that are essential for better prediction and mitigation of long-term effects. Remediation of marine oil contamination is extremely difficult and most often involves dispersal by chemical spraying or physical removal of oil slicks, whereas larger fractions of the crude oil evaporate, dissolve, form oil-in-water dispersions, sediment and/or are biodegraded before removal by human intervention^24^. Rapid decision on mitigation interventions is therefore crucial to avoid long-lasting consequences of oil spills on marine ecosystems.

The goals of this study were thus to measure the earliest ecotoxicological effects (first days) of oil spills on marine biota, and additionally to test a number of tools that may enable rapid assessment of the biological availability and potential ecotoxicological effects of oil dissolved or entrained in marine water. Sample oil content can be broadly estimated by *in situ* or on-board deployment of fluorometry or hexane-extracted total attenuated reflection infrared spectroscopy, whereas, unfortunately, most advanced analytical and ecotoxicological assays take weeks or longer to be carried out and are rarely field compatible. A number of potentially rapid bioassays for oil bioavailability have been developed^25^,^26^,^27^ but have never been deployed directly on board. Assessing their usefulness might provide oil responders with better tools to measure the extent of oil contamination in the field and more appropriately direct their clean-up or mitigation strategies.

Essentially the three types of experiments were collectively performed during two short cruises on the North Sea in a unique constellation of marine biologists, chemists, ecotoxicologists and oil responder teams. The most important type of experiment, but the most difficult to control, consisted of two supervised and authorized spills of 4–5 m^3^ crude oil on open sea in 2008 and 2009, and immediate monitoring of the spill fate during the subsequent days. Two different types of crude oil were used for the supervised spills, since different physical properties and chemical compositions can lead to different behaviours and extent of pollution in the marine environment^12^. To circumvent the relatively unpredictable nature of a sea surface experimental oil spill and also to calibrate and extrapolate effects to a more intense spill, the second type of experiment was conducted on board using 1-m^3^ volume cube vessels that were filled with natural seawater from the same location but that received relatively high doses of crude oil. The third type of experiment consisted of sampling and analysis of seawater nearby and below visible oil slicks encountered along the major North Sea shipping routes, which we assumed had arisen as a result of bilge water releases or tank washing discharges. Using a variety of on-board and remote laboratory methods ranging from standard chemical analysis^12^, bacterial^27^ and mammalian cell line bioreporter assays^28^, we find that dissolved and dispersed oil components become available to marine biota within a few hours after and even at a depth of 8 m below the spill. Algal bioassay^29^ and analysis of various marine plankton variables^30^,^31^ indicate immediate biological effects of the dissolved spilled oil in natural seawater, and this becomes aggravated at higher concentrations such as simulated in the mesocosms.

## Results

### Open sea spills

**Figure 1:**
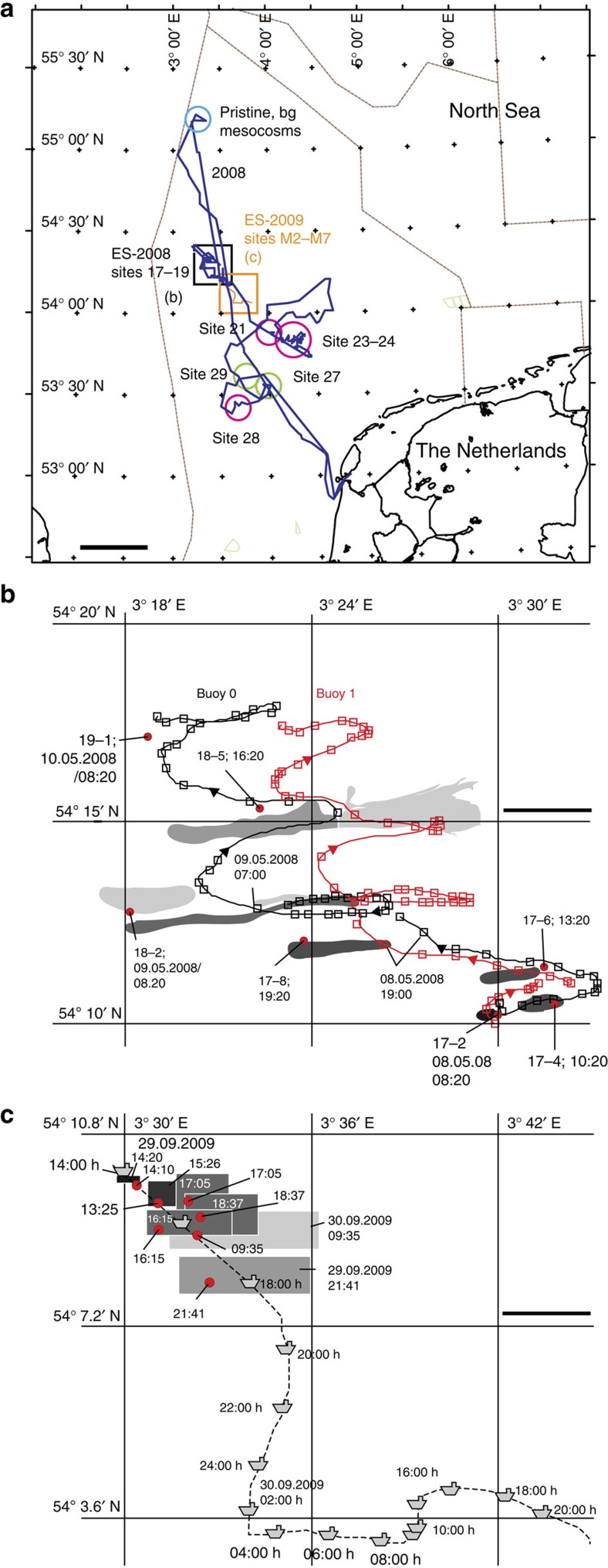
Overview of the experimental and accidental open sea spill locations on the North Sea. (**a**) Comparative locations of experimental spills (ES) ES-2008 (sites 17–19) and ES-2009 (sites M2–M7), the location of sampling of the pristine seawater for the mesocosms and for background (bg) controls, and further sampling sites (21, 23–24 and 28, with visible slicks, magenta; sites 27 and 29, no visible slicks, green). (**b**) Enlargement of the ES-2008 area with the location of the sampling sites, the visual aspect of the slick development (in grey: lighter shades representing thinner oil sheens on the surface) and the traces of both tracking buoys (red and grey squares). (**c**) ES-2009 with the schematic aspect of the slick (grey) over time (red, observation points), and the location of the research/survey vessel Arca (boat symbol). Times in MET. Scale bar, 50 km (**a**); 3 km (**b**,**c**).

**Figure 2:**
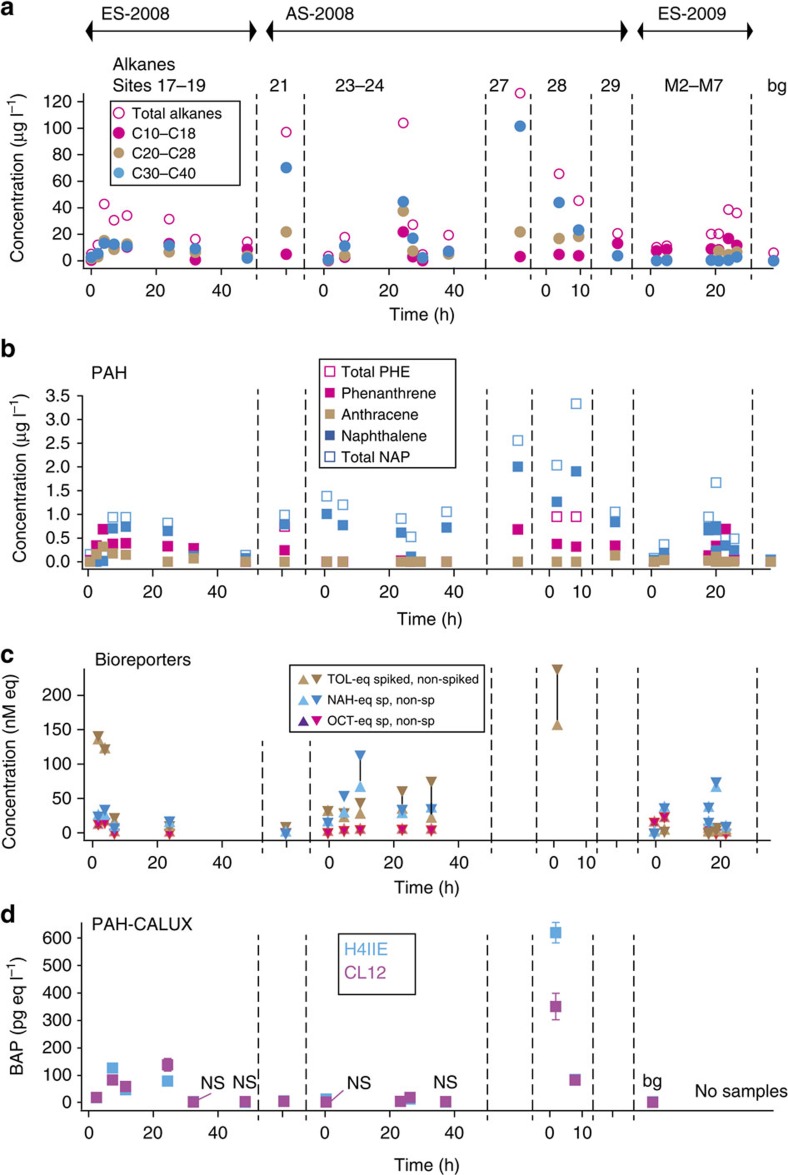
Overview of dissolved oil compound concentrations measured in both experimental (ES) and accidental spills (AS) at −1.5-m depth. (**a**) Total extracted and summed alkane concentrations by chemical analysis. (**b**) Total extracted selected polycyclic aromatic hydrocarbon (PAH) concentrations (NAP, naphthalenes; PHE, phenanthrenes) by the chemical analysis. (**c**) Bioavailable alkane (OCT-eq, essentially C_5_–C_10_ linear alkanes), toluene (TOL-eq, essentially the group of toluene, benzene, ethylbenzene, *m*- and *p*-xylene) and naphthalene (NAH-eq, essentially naphthalene, phenanthrene and mono-methyl derivatives) concentrations (in nM compound-equivalent) measured in on-board bacterial bioreporter assays^27^. High and low values correspond to averages determined from triplicate assays spiked or not with 50–100 nM pure standard (variations from triplicate assays is 5–10% of the arithmetic mean). (**d**) Benzo[*a*]pyrene (BAP) concentrations (pg l^−1^) equivalent to the response elicited by seawater samples in the rat hepatoma cell line-based PAH-CALUX toxicity assays (H4IIE and CL12 (ref. 28)). Data points are the mean from triplicate assays on the same sample. Error bars denote calculated s.d.'s. NS, nonsignificantly different from seawater background (bg), average from three ES-2008 pristine locations. Note that time axis for ES is the time after spill release; for AS it means the time after encounter of the spill.

Two independent deliberate experimental spills (ES) with 4–5 m^3^ crude oil of different composition (Supplementary Table 1) were carried out under supervised conditions in an area in the Netherlands Exclusive Economic Zone of the North Sea (Fig. 1a; Supplementary Table 2). The slicks in the experimental spills remained visible for 3 days in Spring 2008 and ∼1 day in Fall 2009, as a result of more windy conditions (4–5 bft) and higher waves (0.5–1 m) than in 2008, leading to faster breakup of the slick^32^. Both slicks covered a surface of up to 8 km^2^ before dissipating from the surface (Fig. 1b,c; Supplementary Fig. 1). The early compositional changes of the oil slick itself arising from hydrocarbon evaporation and dissolution during the 2009 supervised spill were the subject of a recent separate publication^12^. Chemical analyses of commonly assessed dissolved and dispersed (for simplicity throughout the text we only use the term *dissolved* to denote both) oil compounds in the seawater column by total extraction showed that total *n*-alkanes below the slick reached concentrations of almost 40 μg l^−1^ (Fig. 2a; Supplementary Tables 3–5). Maximum total alkane concentrations were reached after 10 h in 2008 and 24 h in 2009 (Supplementary Fig. 2). Toluene concentrations in the water column reached 100 μg l^−1^ at 1.5-m depth 17 h after the 2009 spill, and even at 8-m depth toluene concentrations of as high as 33 μg l^−1^ were measured after 19 h (ES-2009, Supplementary Table 5). Maximum concentrations of naphthalene and phenanthrene both in ES-2008 and ES-2009 at 1.5-m depth varied between 0.3 and 0.7 μg l^−1^, whereas most other polycyclic aromatic hydrocarbons (PAHs) were present in lower concentrations (Fig. 2b; Supplementary Tables 3–5). Dissolved compound concentrations in the deeper layers (3 and 8 m) followed those in the top layer (1.5 m) almost immediately, indicating rapid vertical distribution through the water column, consistent with a model for density-driven vertical mixing^12^ (Supplementary Figs 3 and 4). Whereas surface layers (1.5-m depth) showed signs of decreasing concentrations after 24 h, dissolved compound concentrations showed no decrease in the deeper water (8 m depth) within this time (Supplementary Figs 3 and 4). Measurements on the ES-2009 spill indicated that even when the slick had visibly disappeared after ∼20 h, concentrations in the seawater column remained high, and dissolved oil compounds had spread from the water body directly underneath the slick to distances of at least 500 m (Supplementary Fig. 2b; Supplementary Tables 4 and 5 ‘outside slick').

On-board bioreporter assays using bacterial strains engineered to produce bioluminescence on recognition of specific compound groups^27^ showed clear responses in the earliest spill samples (2 and 6 h), indicating that dissolved oil components were immediately available to biota (Fig. 2c). Oil bioavailability in samples over time followed the trends of chemically determined dissolved oil components (Supplementary Fig. 3). Bacterial bioreporters specific to toluene and related monoaromatic compounds responded with bioluminescence equivalent to 150 nM (nM-eq, 14 μg l^−1^) dissolved toluene at 1.5-m depth 3 h after the spill in ES-2008 before decreasing, and almost constantly up to 9 nM-eq (1 μg l^−1^) for 24 h in ES-2009 (Supplementary Fig. 5). Short-chain (C_5_–C_10_) alkane-specific reporter bacteria revealed the appearance after 4 h of up to 16 nM-eq (2 μg l^−1^) dissolved octane at 1.5-m depth seawater in ES-2008 before declining to undetectable levels after 24 h. Dissolved octane rose up to 24 nM-eq (3 μg l^−1^) with slow decline in ES-2009, as expected for dissolving and subsequently weathering oil. Bioreporters specific for naphthalene signalled up to 35 nM-eq (4.6 μg l^−1^) after 4 h at 1.5-m depth in ES-2008 with slow decrease in the next 24 h, but produced values of up to 75 nM-eq (10 μg l^−1^, 8-m depth) in ES-2009 seawater even after 20 h (Fig. 2c; Supplementary Fig. 5). Bioreporter analysis of the 8-m depth samples in ES-2009 showed immediate and constant contamination of the deeper water layer, confirming data from chemical analysis, with even higher naphthalene-equivalent concentrations than in the surface layer (Supplementary Fig. 5).

Significant toxicity using the rat hepatoma cell line-based CALUX-assay was detected in seawater samples from the ES-2008 spill at 1.5-m depth during the first 24 h, with values reaching up to an equivalent of 150 ng l^−1^ benzo[a]pyrene (BAP-eq, Fig. 2d). In contrast, no significant differences in an algal viability bioassay based on intracellular esterase activity with the algae *Micromonas pusilla* as model species were observed between samples from under the ES-2008 oil slick and from non-polluted control sites (Supplementary Fig. 6).

Total unicellular phytoplankton abundances were not significantly different between oil-contaminated water and control locations, independent of phytoplankton group, type of spill and sampling depth (Supplementary Figs 7 and 8). However, average chlorophyll-*a* (Chl-*a*) concentrations as indicator of algal biomass were slightly lower in samples taken after 1 day 1.5 m below the oil slicks of ES-2008 (91 ng l^−1^) than at the same depth outside visible spills (128 ng l^−1^, *P*=0.064, one-tailed *t*-test, *n*=2, Supplementary Fig. 9). Furthermore, primary production in samples taken at 1.5 m below slicks was significantly lower than from those outside the slick (analysis of similarity (ANOSIM), Euclidian distance, *n*=11, *R*=0.26, *P*<0.05, Supplementary Fig. 10). Pigment analysis showed that Chrysophyceae, Prasinophyceae and Prymnesiophyceae were the dominant algal groups in the water (Supplementary Fig. 11). Particularly, Eustigmatophyceae and Cryptophyceae were sensitive to the oil and had disappeared already within 2 h from samples underneath the ES-2008 slick compared with outside spill controls (Supplementary Fig. 11). Heterotrophic bacterial abundances were not significantly different between samples from underneath and outside the oil slick (on average 8.8 × 10^5^ cells per ml); neither were the relative proportions of ‘high'- and ‘low-DNA' bacteria^33^ among the heterotrophic community (on average 47% and 53%, respectively). Average viral abundances were also the same in samples from below and outside the oil slick (4.7 × 10^7^ particles per ml).

In contrast, heterotrophic nanoflagellates reacted particularly sensitively to dissolved oil in the seawater column. Their average number was more than threefold lower in samples 3 days after the oil release from below the ES-2008 slick compared with the same depth (3 m) outside visually contaminated zones (365±59 versus 113±15 cells per ml, respectively; two-tailed *t*-test: *P*=0.006, *n*=6). During the ES-2009 spill, the average number of heterotrophic nanoflagellates 1.5 m below the slick after 20 h was 1.8-fold lower compared with that outside the oil slick (271±180 and 488±126 ml^−1^, respectively). In addition, microzooplankton ciliates at 1.5-m depth declined more than twofold 2 h and 1 day after the ES-2009 spill (∼13 ml^−1^ inside versus ∼35 ml^−1^ outside slick). Zooplankton larvae declined threefold within 1 day in samples underneath the slick; however, the total number counted was low (<10). Tintinnids at 1.5-m depth declined to 57–80% of their abundance within 1 day compared with outside slick seawater.

### On-board mesocosms

**Figure 3:**
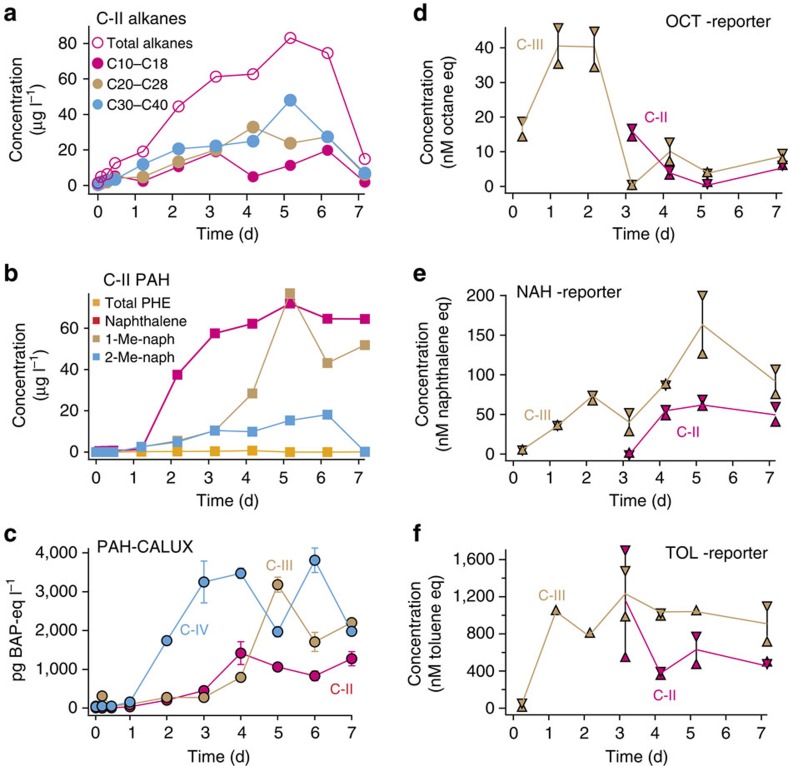
Mesocosm dissolved oil compound concentrations. (**a**) Total extracted selected summed alkane concentrations by chemical analysis over time in mesocosm C-II (2 l crude oil dosed per m^3^) measured at the bottom tap (25 cm above container floor). (**b**) Total extracted selected polyaromatic hydrocarbon (PAH) concentrations by chemical analysis. Total PHE, sum of phenanthrene and methylphenanthrenes. 1-Me- and 2-Me-naph, 1- and 2-methylnaphthalene, respectively. (**c**) Toxicity equivalent of pg benzo[*a*]pyrene (BAP) per litre in bottom-sampled mesocosm vessels (C-II; 2 l and C-III, 5.5 l crude oil dosed per m^3^; C-IV, 2 l crude oil but ultraviolet treated) in the rat hepatoma cell line-based PAH-CALUX bioassay^28^. Data points are the mean from triplicate assays on the same sample. Error bars denote calculated s.d.'s (**d**–**f**) Bioavailable alkane (OCT-eq, essentially C_5_–C_10_ linear alkanes), toluene (TOL-eq, essentially the group of toluene, benzene, ethylbenzene, *m*- and *p*-xylene) and naphthalene (NAH-eq, essentially naphthalene, phenanthrene and mono-methyl derivatives) measured in the bacterial bioreporter assays. Concentrations expressed as nM equivalent standard. High and low values correspond to averages determined from triplicate assays spiked or not with 50–100 nM pure standard (variations from triplicate assays is 5–10% of the arithmetic mean). d, days.

To calibrate bioreporter responses and better interpret effects observed on plankton in the outside spills, we simulated a more intense oil spill in two 1-m^3^ mesocosm (C-II and C-III) experiments placed on board the research vessel with two others (C-I, no oil, and C-IV, ultraviolet sterilized) serving as controls. Linear alkanes (above nC_10_) and PAHs gradually dissolved in the aqueous phase, with alkanes dissolving more rapidly than PAHs (Supplementary Fig. 12). Dissolved concentrations of up to 80 μg l^−1^ for total alkanes and naphthalene were detected, which is 2 and 30 times higher than observed in the open sea spills, respectively, confirming the higher intensity of the mesocosm spill (Fig. 3a,b). Because the mesocosm containers were closed most of the day (18–20 h) to avoid evaporation, not only the concentrations but also the proportions of the different PAHs and alkanes were different in the mesocosm seawater compared with that of outside spill (ES-2008, both contaminated with Arabian light; Supplementary Tables 3 and 6). Some contamination was measured in the negative control (C-I, total alkane and total PAH concentrations of 1.5 and 0.16 μg l^−1^, respectively), suggesting a background contamination level even in so-called pristine seawater. There was no obvious difference in dissolved concentrations of alkanes or PAHs between the C-II (2 l crude oil) and C-III (5.5 l) mesocosms (Supplementary Fig. 12).

Bacterial bioreporters detected high concentrations of bioavailable alkanes and toluene in samples taken 1 day after release of the oil in the mesocosms, reaching up to equivalents of 40 nM (4.5 μg l^−1^) and 1.2 μM (109 μg l^−1^), respectively (Fig. 3d,f). This is three (for alkanes) and eight (for toluene) times higher than observed in the open sea experimental spills, and confirm the aggravated effects on biota (Supplementary Table 6). Bioavailable naphthalene-equivalent concentrations reached 150 nM (20 μg l^−1^) after 5 days, which is four times higher than for samples from ES-2008 with the same crude oil (Fig. 3e).

**Figure 4:**
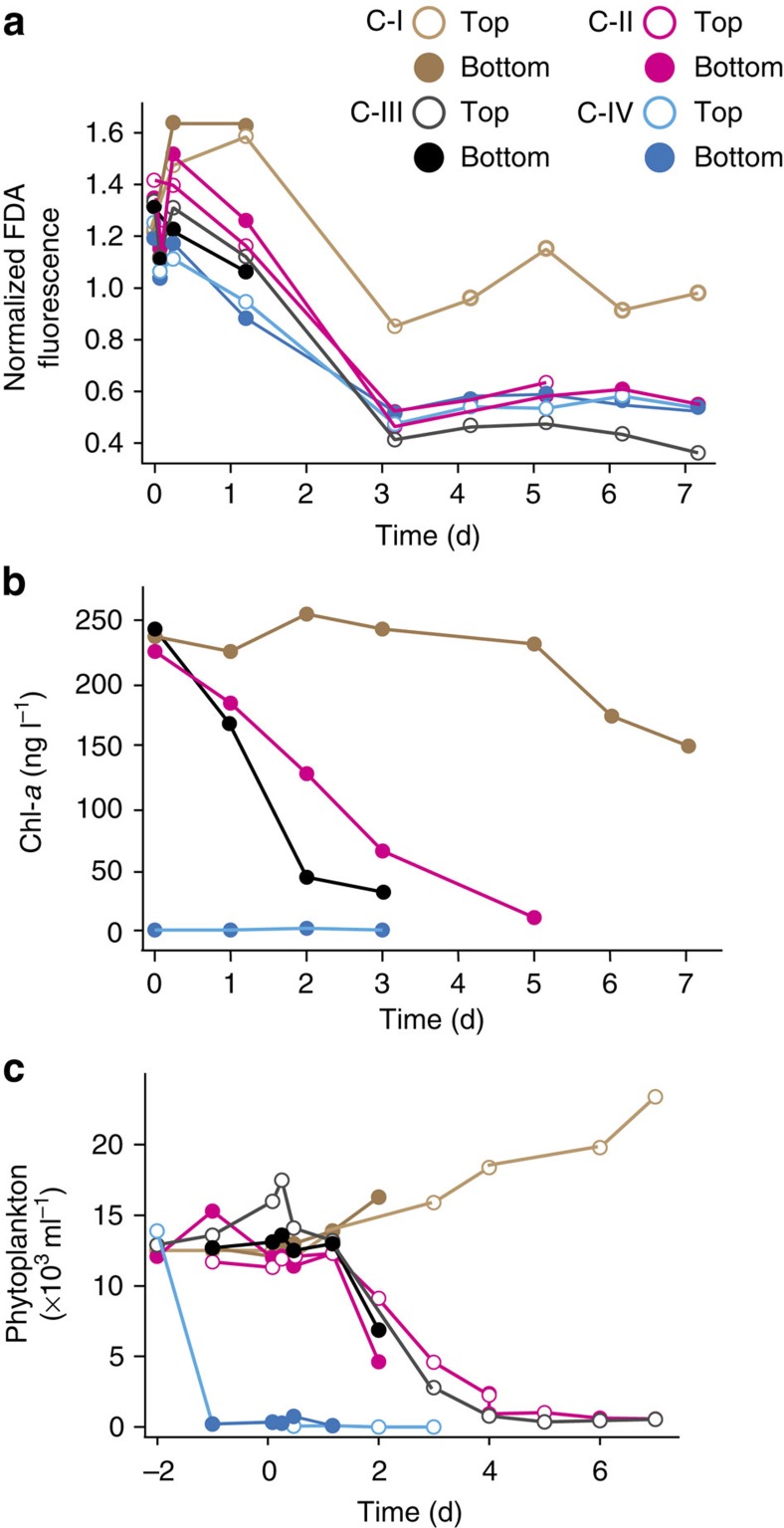
Phytoplankton responses to oil pollution in the mesocosms. (**a**) Viability of *Micromonas pusilla* exposed to mesocosm samples, expressed as fluorescence released from stainings with fluorescein diacetate (FDA) normalized to an incubation in control medium (=1). (**b**) Total chlorophyll-*a* (Chl-*a*) concentrations (ng l^−1^) over time in the mesocosm vessels. (**c**) Total phytoplankton abundance (flow cytometric analysis, cells<20 μm) over time in the four mesocosms at the top (open symbols) and bottom (closed symbols) of the containers. Mesocosm indications: C-I, non-contaminated; C-II, 2 l crude oil per m^3^; C-III, 5.5 l crude oil per m^3^; C-IV, 2 l crude oil, ultraviolet treated. Note that time=0 refers to the moment of oil addition. UV treatment of C-IV was done at t=–2 d. top (70 cm above container floor) and bottom (25 cm) refer to sampling ports on the mesocosm vessel. d, days.

CALUX tests indicated BAP-eq concentrations of between 206 and 315 pg l^−1^ already 6 h after the spill in the mesocosms, reaching ∼4 ng BAP-eq per litre after 6 days (Fig. 3c), which is 20 times higher than in the ES-2008 outside spill (Fig. 2d). The gradual increase of toxicity largely paralleled increasing PAH concentrations (Fig. 3b,c). The ultraviolet-irradiated control mesocosm (C-IV) showed a more rapid increase of BAP-eq signal than mesocosms C-II and C-III (Fig. 3c). The *M. pusilla* algal bioassay showed a distinct loss in viability of the algae exposed to samples from contaminated mesocosms after day 1 and thereafter (Fig. 4a). Samples from the non-contaminated mesocosm (C-I) also caused some loss of *M. pusilla* viability, but not before day 3 and much less pronounced than with oil-contaminated samples (Fig. 4a).

Oil pollution in the mesocosms resulted in rapid decline of Chl-*a* concentrations, to ∼50% of the starting concentration for C-II and <20% for C-III within 2 days (Fig. 4b), which was accompanied with a similar decrease in ^14^C-bicarbonate photosynthesis rates (Supplementary Fig. 13). Also, total abundance of phytoplankton decreased by ∼50% 2 days after the spill in mesocosms C-II and C-III, and phytoplankton disappeared almost completely within the next few days (Fig. 4c). Disappearance was mainly a result of decrease in various groups of eukaryotic phytoplankton (Supplementary Fig. 14). As expected, Chl-*a* concentrations and phytoplankton cell abundances in the ultraviolet-treated C-IV mesocosm were zero throughout the entire experiment (Fig. 4b; Supplementary Fig. 14), whereas in the uncontaminated mesocosm they remained more or less constant and high (Fig. 4b,c, brown points). As a result of slower decline of cyanobacteria (*Synechococcus* spp.) than the eukaryotic phytoplankton groups (Supplementary Fig. 14), they became the dominant group of photoautotrophs after 3 days in the oil-contaminated mesocosms (Supplementary Fig. 15). Similar to the ES-2008 spill, the Eustigmatophyceae disappeared from oil-contaminated mesocosms, but also Chrysophyceae declined to undetectable level (Supplementary Fig. 15). In contrast, Dinophyceae seemed to better resist oil pollution than other eukaryotic phytoplankton groups. The number of tintinnids in the C-II and C-III oiled mesocosms strongly decreased within 2 days to 16% of that in C-I, and remained low for the rest of the experiment.

### Accidentally encountered spills

During the 2008 campaign, we also sampled and analysed a number of accidentally encountered slicks (sites 21, 23–24 and 28; Fig. 1a) along with two visibly non-contaminated sites (sites 27 and 29; Fig. 1a; Supplementary Table 2) nearby the major shipping route on the North Sea. The spill encountered at sites 23–24 apparently was a recent unsupervised spill or else an undocumented natural oil seep, for which it was possible to follow the dissolution and disappearance of oil compounds over time (Fig. 2). Although we do not know the history of these accidentally encountered spills (that is, date of spilling, and type and quantity of oil spilled), the concentrations of alkanes and PAHs were occasionally even higher than in the experimental spills. For example, site 21 showed comparatively very high C_30_–C_38_ concentrations, whereas site 28 showed higher PAH concentrations (Fig. 2; Supplementary Table 3). Similar to experimental spills, the dissolved compound concentrations detected in the deeper water layers (3- and 8-m depth) were comparable to just below the slick (1.5 m; Supplementary Figs 3 and 4), again indicating rapid distribution. Disturbingly, seawater from both sites 27 and 29, which had no visual aspect of oil contamination, contained significant alkane and PAH levels comparable to seawater samples taken directly from below accidental oil slicks (Fig. 2a,b). This suggests that seawater near the major shipping routes on the North Sea is recurrently contaminated with oil, either from unintentional spills or maybe from natural oil and gas seeps^34^,^35^.

Bacterial bioreporter and CALUX toxicity assays showed bioavailability of oil compounds that were largely comparable to the supervised spills. The accidental spill at site 28 showed high toxicity with values up to 320 pg l^−1^ BAP-eq, and toluene equivalent concentrations of up to 235 nM (21 μg l^−1^; Fig. 2d). The 23–24 spill samples showed significant PAH concentrations as well as bioavailable alkanes, toluene and naphthalene (up to 100 nM-eq), but comparatively low CALUX toxicity (between 3 and 14 pg l^−1^ BAP-eq, Fig. 2d). Seawater from below spill 21 showed bioavailable concentrations of alkanes, toluene and naphthalene close to the detection limit (1 nM-eq) despite relatively high chemically measured alkane concentrations.

The algal *M. pusilla* viability assays as well as the phytoplankton community parameters (abundances, Chl-*a* and primary production) were not significantly different with samples from underneath or outside slicks, although occasionally nanoeukaryotic phytoplankton abundances were decreased in samples underneath the slicks (for example, spills 21–24, Supplementary Figs 7 and 8).

## Discussion

Despite small open sea spills producing a seemingly small and transient visual presence of floating slick, our results from both the experimental and accidental spills show that almost instant contamination occurs of a large water body below (down to 8 m at least) and away (up to 500 m away) from the visible slick. Contamination to such depth is surprisingly rapid and merits to be studied further. Concentrations of dissolved oil compounds below such slicks provoke clear biotic effects, attested by the results from bacterial and mammalian bioreporter assays. Reporter cell output followed both qualitatively and quantitatively the chemically measured trends from oil compounds dissolving in seawater (for example, Supplementary Figs 2–5), and thus substantiate how organisms in the aqueous phase become exposed to the dissolved oil. Disturbance of sensitive plankton species was also detected within 1 day after the spills. Observed trends intensified in on-board seawater mesocosms receiving comparatively higher oil dosages, which corroborates the more moderate biological effects detected in sea spills. Although the mesocosm spills are a bit artificial in the sense that they are largely closed to the open atmosphere, they were useful to calibrate the bioreporters and construe plankton effects. In themselves they might be representative for larger oil spills or spills in more confined areas, such as oil trapped under ice or in a bay, spills in windless, quiescent sea surface conditions, where evaporation rates are decelerated, or deep-sea releases where the emitted oil does not immediately contact the atmosphere (thereby also suppressing evaporation). It is to be expected that the early effects as documented here are also relevant for large spills.

At the risk of making oversimplifications, we argue that any other living organism present in the same water body is exposed to available oil compound concentrations similar to those eliciting clear toxicity responses in the reporter cells. Bacterial and PAH-CALUX mammalian reporter assays, which consist of living cells producing luciferase in response to the presence of specified target compound class^27^,^28^, per definition respond only to bioavailable compounds, that is, compounds interacting with or entering the cells during the incubation period of the assay^36^. The bacteria are engineered to respond when short-chain linear alkanes, monoaromatic solvents such as toluene or low-molecular-weight PAHs, enter the cell^27^. Responses in the PAH-CALUX assay are due to the action of high-molecular-weight PAHs on Ah receptor-mediated metabolic activation and have been shown to predict the presence of carcinogenic PAH mixtures^28^, and elevated levels of such compounds clearly can lead to long-term health effects in humans and other mammals^37^. Extrapolation from bioreporter signals to longer-term effects is obviously difficult, because pharmacokinetics can differ widely between organisms, due to differences in uptake and excretion rates, and alleviation by metabolism, detoxification and repair mechanisms. Effects may also depend strongly on whether organisms have a motile (for example, fish) or sessile life style, and can mitigate potential toxicity by migration to cleaner water masses. However, bioreporter data clearly show the toxicity of the seawater below small spills, and thus particularly areas where small spills are occurring frequently are likely to lead to chronic effects on marina biota.

Not only reporter cells exposed *ex situ* to seawater samples but also analysis of natural seawater plankton confirmed *in situ* biological effects of the dissolved oil. Several natural zooplankton groups (heterotrophic nanoflagellates, tintinnids, ciliates and copepod larvae) seemed particularly sensitive to immediate exposure at the dissolved oil concentrations found underneath the experimental spills and their numbers decreased >50% within 1 day. Given the large volume of contaminated water, it seems unlikely that zooplankton numbers decreased so rapidly due to migration out of the contaminated water body. The susceptibility of dominant groups of zooplankton to dissolved oil compounds is in agreement with other studies^38^,^39^ and may cause a release of grazing pressure on phytoplankton^40^, which may result in increased biomass of specific algal species within a few days after the spill. Also, certain sensitive algal species groups (notably, Eustigmatophyceae) disappeared even after short (2 h) exposures (Supplementary Fig. 11). It should be kept in mind, however, that the North Sea is subject to frequent small oil releases from shipping activities and maybe even from natural seeps^35^. Therefore, we cannot exclude that the plankton community in our control samples outside the slick zones had actually been exposed earlier to another contaminated water body. No effects were found on heterotrophic bacterial abundance and also previous studies did not see evidence for oil-degrading bacteria developing within such short time periods^41^. Oil-contaminated seawater in mesocosms immediately and strongly reduced overall Chl-*a* levels, algal viability (*M. pusilla* viability test) and phytoplankton community composition (various phytoplankton groups disappeared) with direct effects on photosynthesis (Supplementary Fig. 13). This confirmed the weaker trends observed for the outside spills. Toxicity of oil to phytoplankton is widely species dependent, affecting different cellular and molecular targets^42^, but the mesocosms indicated significant changes to be expected at the base of the food chain within the first few days after a more intense oil spill, which are likely to impact ecosystem productivity. In particular, the loss of zooplankton predators and the relative resistance to oil of cyanobacteria and autotrophic dinoflagellates contribute to the change in algal composition, and suggest ecological changes to occur faster and deeper in the water column than thus far anticipated.

How do our results relate to other (eco)toxicological studies on marine oil contamination and how likely are short exposures (1–3 days) lead to long-term effects? This is obviously a difficult projection given the huge variety of crude oils and will certainly underestimate the contribution to the sample toxicity of the many thousands of (un)characterized compounds in oil. However, one possibility for comparison is to rely on certain reference compounds, such as linear alkanes or PAHs, which have frequently been measured, without implying that sample toxicity is solely due to those reference compounds. The chemical and bioavailable concentrations of reference alkane, monoaromatic and PAH compounds detected in our North Sea spills and on-board mesocosms incubations are lower than what has been used in a variety of acute-toxicity studies^6^,^14^,^43^,^44^,^45^ but comparable to others^5^,^46^, allowing some extrapolation of potential effects despite the general limitations emphasized above. For example, fish juveniles displayed cardiac dysfunction when exposed to samples taken from the Deepwater Horizon blowout^5^ due to impaired excitation–contraction coupling^47^. Seawater samples from within the Deepwater Horizon plume contained total PAHs in a concentration range of 1–15 μg l^−1^ (ref. 5), and monoaromatic hydrocarbons in excess of 50 μg l^−1^ (ref. 6), which is similar to concentrations that we found in peak spill samples and mesocosms (Figs 2, 3; Supplementary Tables 3–5). Also, copepod assays measured 30% of individuals displaying active avoidance during a 12-h period of exposure to oil-contaminated water samples with a reference concentration of 19 μg l^−1^ total PAHs, whereas lethality occurred at a 24-h median to samples containing total PAH concentration of 43 (26–92) μg l^−1^ (ref. 46). Further effects of oil exposure to marine biota under similar reference compound concentrations included long-term growth effects, increased mortality and behavioural dysfunction^43^,^44^,^45^. Because of the similar reference compound ranges between such studies and ours, we believe it is likely that oil-contaminated seawater samples in the early phases after a spill and even for the duration of a small spill (1–3 days) provoke physiological dysfunctions of copepods, larval and embryonic fish.

Given the different compositions of both oils (Supplementary Table 1) and the changing composition of individual oil components in the seawater phase over time, it is interesting to get an approximate idea of whether the concentrations measured in the seawater samples at different moments corresponded to what one would expect from the total possible dissolvable compound mass of the released volume of oil. We estimated slick coverage and corresponding contaminated seawater volume for a total depth of 8 m, using the time point of highest measured concentrations in the seawater by both chemical analysis and bacterial bioreporter assays (Table 1). For ES-2008, this produced reasonable estimates with between 0.5% of C_5_–C_10_ linear alkanes (from bioreporter analysis) and 141% of total alkanes (from chemical analysis) being released into the water column. However, for ES-2009, the estimates based on chemical concentrations in the water phase were much higher than what would be possible from available compound mass in the released oil volume. Similarly confounded field mass balance results have been obtained based on other water column sample measurements reported in a previous study of the ES-2009 spill, using a different sampling technique^12^. However, it should be considered that field mass balance calculations are prone to substantial errors resulting from measurements on a very limited number of discrete samples. Consistent with these mass balance estimates, previous mass transfer model calculations^12^ also predict that water column concentrations of these hydrocarbons should be much lower than what is apparently measured both by chemical analysis and bioreporters, for the ES-2009 spill. The higher than expected water column concentrations maybe due to rougher weather conditions (stronger mixing of surface waters) that may have resulted in inclusion of oil microdroplets in the water phase before total chemical extraction analysis as suggested by previous experimental data and models^48^, or to mixing with another nearby seawater body that had recently been contaminated through anthropogenic activity or from natural seepage. Although the possibility of external contamination seems remote, it is supported by the alarmingly high total alkane and PAH concentrations in seawaters at two locations (sites 27 and 29; Fig. 2a,b) without visual oil contamination.

In conclusion, and contrary to popular belief, our study testifies that even small oil spills rapidly (few hours) result in bioavailable exposures with potential toxic effects. The fact that intensive shipping zones such as the North Sea show a multitude of such small ‘accidentally' encountered spills and even contamination of seawater without visible oil slicks (that is, sites 27 and 29) indicates to us that frequent short-term and chronic exposure of available and toxic compounds to eukaryotic micro- and macroorganisms occurs with potential long-term adverse effects. Distinct differences in sensitivity to dissolved oil were observed among the phyto- and zooplankton groups, which follow-up studies should attempt to dissect at molecular and mechanistic level. Our results and bioassays are also very relevant for larger oil spills, in particular concerning the initial temporal aspects after the oil release, as this is typically not monitored. With surging demands for marine oil exploration the frequency of small spills is likely to increase rather than decrease, and thus poses a serious risk to marine ecosystem health. This calls for better *in situ* and real-time monitoring of marine ecosystems for dissolved oil compounds, which *inter alia* we demonstrate could be achieved by bacterial and mammalian bioreporter assays, or with algal viability assays employing more sensitive species than the currently used *M. pusilla*^29^. Such tests are relatively simple and can be used on board of vessels or, possibly, in automated monitoring stations, and their results can help to decide rapid and appropriate immediate mitigation actions, even in the case of frequently occurring small oil spills.

## Methods

### Oil spills and sample collection

On two separate occasions, from 8–10 May 2008 to 29–30 September 2009, an experimental oil spill was conducted on the North Sea using research vessel Pelagia (2008) and research/survey vessel Arca (2009), under permission of and in collaboration with the Netherlands Ministry of Transport, Public Works and Water Management, Rijkswaterstaat Noordzee (RWS-NZ). The experimental area occupied a zone in the Netherlands Exclusive Economic Zone, that is, 54° 17′ N and 3° 50′ E for the 2008 spill, and 54° 10′ N and 3° 30′ E for the 2009 spill (Fig. 1). The sites were selected for their location far off the coast, minimum fishing activity and relatively low bird abundance. Details of the 2008 experimental and sampling strategy have been reported previously^49^. Temperature of the seawater was around 12 and 16 °C for 2008 and 2009, respectively. The 2008 experimental spill consisted of 5-m^3^ Arabian Light crude, mixed with 5 kg rhodamine in 1-m^3^ seawater to assist in visualization. On the basis of its high aqueous solubility, we assumed that the rhodamine acted as a tracer for the most water-soluble hydrocarbon compounds, as they migrate through the water column. Two tracking buoys (Fig. 1b) served to follow and locate the ES-2008 spill. The 2009 spill was conducted with 4-m^3^ Grane crude from Norway without addition of rhodamine (see Supplementary Table 1 for oil characteristics). No tracking buoys were deployed in ES-2009, and Fig. 1c and Supplementary Table 2 report the location of the vessel and oil slick, but not the exact sampling site. The nature and composition of the oils released at the accidental spill sites were not known.

The seawater below the visible spill zone (at 1.5-, 3- and 8-m depth), at the edge (2009) and outside the visible slick (500-m distance) was sampled from a rubber boat using a specific device^25^, which consisted of a metallic tube attached to a 25-mm diameter hose, connected to a membrane pump on board of the rubber boat (10 l min^−1^ at 1.5 bar)^25^. The metallic tube was opened and closed from a distance and passes through a water-filled bucket to avoid contamination of the tube end with the oil. The first 10 l of seawater for each sample was discarded, after which the sample was collected in metal containers or in 20-l glass bottles, protected from light and filled to the top to minimize evaporation of oil components. Seawater outside the slick was sampled at a distance of at least 500 m from the visible slick using Niskin bottles mounted on a metal frame equipped with conductivity, temperature and depth sensors (CTD) (Seabird 9+; with standard sensors and auxiliary sensors for chlorophyll autofluorescence, Chelsea Aquatracka Mk III). Samples were transported back to the vessel within 30 min and immediately dispatched for further on-board analyses. Subsamples of 0.5 l were frozen at −20 °C and transported in frozen state to laboratories on the shore for toxicity analyses (see below).

### Mesocosm incubations

During the 2008 campaign, four 1.25 × 1 × 1 m (*w*, *l*, *h*) plastic cube vessels (mesocosms) were filled with 1-m^3^ natural seawater (from the same location as the 2008 experimental spill) and incubated on the aft deck under *in situ* subsurface light conditions. The vessels were pretreated with 0.1 N HCl solution (filled to the top) for more than a month (placed outside), after which they were thoroughly rinsed with seawater. These mesocosms either received no further amendment (control cube vessel, C-I), 2 l of Arabian Light (C-II) or 5.5 l of Arabian Light (C-III). Finally, one mesocosm filled with pristine seawater was treated first with ultraviolet light to kill biological activity, after which 2 l of Arabian Light was added (C-IV). Cube vessels were covered with a 30-cm ø cap, which was unscrewed a few times per day for 1–2 h to allow gas exchange. Each vessel was sampled at the top (t, situated 70 cm above the container floor) or bottom (b, 25 cm) through taps that were placed in the side of the cube vessels, for as long as the water surface was within reach of the taps.

### Natural plankton

Samples (3.5 ml) for phytoplankton enumeration were analysed fresh (2008 cruise) or fixed (2009 cruise) using a Becton Dickinson Calibur benchtop flow cytometer (FCM; BD Biosciences, Erembodegem, Belgium), equipped with a 15-mW Argon laser (488-nm excitation). Samples were fixed for 30 min at 4 °C with 1% final concentration paraformaldehyde buffered with hexamine (solution made of 18% (v/v) paraformaldehyde and 10% (w/v) hexamine in water), flash-frozen in liquid nitrogen and stored at −80 °C. Tests during the 2008 cruise showed that counts from fixed samples were comparable to the fresh samples determined on board. Phytoplankton groups were discriminated based on their natural red (chlorophyll *a*) and yellow-orange (phycoerythrin pigment of *Synechococcus* spp.) autofluorescence in combination with side scatter^31^.

Enumeration of prokaryotes and viruses was performed on 1 ml 0.5% glutaraldehyde fixed samples (EM-grade 25%, Aldrich), flash-frozen and stored at −80 °C until analysis by FCM according to ref. 50. In short, thawed samples were diluted with 1 mM Tris-EDTA buffer (pH 8.0) and stained with SYBR Green I in the dark for 10 min at 80 °C for viruses and 15 min at room temperature for bacteria^30^.

Heterotrophic nanoflagellates were counted using epifluorescence microscopy in 20-ml samples fixed with 1% final concentration glutaraldehyde (10% stock concentration) and filtered onto a 0.2-μm black polycarbonate filter (25 mm ø, Whatman). Samples were then stained using 4′6-diamidino-2-phenylindole dihydrochloride (5 mg ml^−1^, Sigma-Aldrich) at a final concentration of 1 μg ml^−1^ and stored at −20 °C, and counted using a Zeiss Axiophot epifluorescence microscope equipped with BP365, FT395 and LP397 excitation and emission filters. Microzooplankton was counted by inverse light microscopy in 100-ml samples that were fixed with Lugol solution and stored at 4 °C until analysis.

### Primary production

Sample (50 ml) was poured in a glass tube with a screw cap and 12.5 μCi ^14^C-bicarbonate was added. Control and oil spill seawater samples were incubated for 2 h in a rotating incubator under 10 different light intensities (0–600 μmol photons per m^2^ per s) with an artificial light source. Samples from each of the four mesocosms were incubated at two light intensities (30 and 72 μmol photons per m^2^ per s). After 2-h incubation, the samples were filtered over glass fiber filter (GF/F) glass fibre filters; the filters were placed 30 min over hydrochloric acid fume to remove any inorganic ^14^C. Finally the filters were put in scintillation vials, stored frozen (−20 °C) and counted with an LKB Rackbeta 1211 scintillation counter after adding 14 ml Instagel. The data (Bq l^−1^) were used to model maximum production (*P*_max_) at the corresponding light intensity (*I*_max_), the initial slope (*s*) and the light inhibition parameter (*w*) according to Eilers^51^.

### Phytoplankton viability bioassay

Changes in the viability of the cultured unicellular marine algal species *M. pusilla* exposed or not to (contaminated) seawater samples were exploited to determine possible oil-related effects^29^. Viability assays consisted of incubating (at 15 °C and 50 μmol quanta per m^2^ per s) 50 μl of exponentially growing *M. pusilla* for 5 h with 1 ml Whatman GF/F-filtered seawater sample, after which fluorescein diacetate (FDA, Invitrogen) was added to a final concentration of 10 μM. FDA is cleaved by intracellular esterases to form fluorescein. At least 1,000 *M. pulsilla* cells per sample were measured by FCM and the mean FDA fluorescence was recorded. Values were normalized against those of control incubations with 1-ml standard *M. pusilla* culture medium.

### Bacterial bioreporter assays

A selection of contaminated seawater samples (mesocosms or outside spills) was assayed directly on board of the vessel in a microbiology safety laboratory container using a set of bacterial bioreporters. The bioreporters consist of living *Escherichia coli* and *Burkholderia sartisoli* cells engineered to produce luciferase after being in contact with a target chemical or group of chemicals^52^). The target specificities included C_5_–C_10_ short-chain alkanes (the ALK reporter); toluene, ethylbenzene and xylenes (TOL reporter); and naphthalene, methylnaphthalene and phenanthrene (NAH reporter). In short, water samples were incubated with an exponentially growing and washed culture of reporter cells for 2 h in 4-ml closed glass vials, after which *n*-decanal was added and the light output was measured in a portable luminometer (Berthold Junior, Berthold AG, Germany). Samples were diluted fourfold with buffer to reduce the seawater salt concentration and then incubated in triplicate assays, directly or spiked with a known concentration of pure target compound (that is, 50 nM octane, 100 nM toluene or 100 nM naphthalene). If necessary, samples were further diluted with artificial seawater before assaying. Light emission was compared with a calibration series in which the reporter cell suspension was incubated with known target compound concentrations at 0, 50, 100, 200 and 400 nM in pristine seawater, carried out simultaneously and incubated for the same duration. Two mean values are reported, those of the sample alone and the spiked sample, corrected for the measured difference of the spiking compared with the expected signal increase according to the calibration curves. Variation of triplicate measurements on the mean was in the order of 5–10%. Values are reported as ‘nM toluene (octane, naphthalene)-equivalent concentration'.

### Toxicity assays using mammalian cell lines

Sample toxicity was further tested in the PAH-CALUX bioassay, which is based on the H4IIe rat hepatoma cell line (source American Type Culture Collection) stably transfected with a *luc* luciferase gene under the control of part of the mouse *CYP1a1* promoter containing dioxin-responsive elements^28^. Frozen samples were transported to the laboratory and thawed 1 day before analysis at 4 °C, and the sample volume was determined by weighing. Samples were extracted with 50 ml hexane and 200 r.p.m. rotary shaking for 1 h at room temperature with the bottles laying on their side. The hexane phase was recovered and transferred to a clean glass tube, and evaporated until a volume of 2 ml. The water sample was extracted with another 50 ml hexane in the same procedure but for 30 min only, after which the second hexane phase was pooled with the first and evaporated to leave 2 ml. This procedure was repeated once more and pooled with the previous. Evaporation was proceeded until 1 ml was left, after which the hexane phase was transferred into a glass point vial, and further evaporated until almost dry. The residues were dissolved in 100 μl dimethylsulfoxide for 30 min and then frozen.

On the day of analysis, the vials were thawed and then further diluted in dimethylsulfoxide at 3, 10, 30, 100, 300 and 1,000 times. Samples and sample dilutions were then incubated for 6 h in triplicate assays in 96-well plates to PAH-CALUX cells, together with a calibration series of known BAP concentrations. Those dilutions giving a measurable response within the calibration range were selected for calculation as described^28^. Values are reported as ‘ng BAP-equivalents per litre'.

### Chemical analyses

Samples for dissolved inorganic phosphate (DIP) and nitrogen (DIN; nitrate, ammonium and nitrite) and reactive silicate (Si) were gently filtered through 0.2-μm polysulfone filters (Acrodisc, Gelman Sciences), after which those for DIN and DIP were stored at −20 °C and those for Si at 4 °C until analysis in the home lab. Nutrient analyses were performed with a Bran+Luebbe Quaatro AutoAnalyzer using established protocols for DIP, DIN and Si. Average dissolved inorganic nutrient concentrations (for all three sampled depths of 1.5, 3 and 8 m) of the natural seawater were 1.13±0.78 μM for DIN (made up of nitrate, ammonia and nitrite), 0.046±0.019 μM for DIP and 1.06±0.67 μM for silicate. Inorganic nutrient concentrations were not statistically different between samples from under oil slicks and visually non-contaminated control locations. DIN and DIP concentrations were also comparable for all mesocosms and were not a particular indicator for changing phytoplankton or bacterial dynamics. Silicate concentrations increased in the oiled mesocosms over time from 0.25 to 0.35–0.40 μM, with highest concentration for the killed C-IV.

Phytoplankton pigment composition was estimated by high-performance liquid chromatography on 1–5-l filtered seawater samples, using 47 mm ø 0.7 μm Whatman GF/F filters at <0.2 kPa, which were stored at –80 °C. Pigments were extracted in 4 ml methanol using glass pearls in a CO_2_-cooled Braun (Melsungen, Germany) homogenizer. Extracts were centrifuged and filtered through 13 mm ø 0.45 μm PTFE syringe filters (Grace Davison Discovery Sciences, Deerfield, IL, USA) to remove cell and filter debris. Pigments were detected at 437 nm with a Dionex photo diode array detector using a Dionex HPLC system equipped with a C_8_ separation column (Luna 3 μ C_8_(2) 100A, 100 × 4.6 mm, Phenomenex, Torrance, CA, USA) thermostated at 25 °C. The solvents used were (a) methanol:acetonitrile:aqueous pyridine (50:25:25, v/v/v) and (b) methanol:acetonitrile:acetone (20:60:20, v/v/v). Standards were obtained from DHI (Hørsholm, Denmark). Results were processed using CHEMTAX^53^.

For PAH analysis, 50 ml of seawater sample was filled into precleaned 70-ml bottles and spiked with 3 μl of a deuterated PAH standard mix, consisting of naphthalene d08 (36.61 mg l^−1^), acenaphthalene d10 (34.62 mg l^−1^), fluorene d10 (34.82 mg l^−1^), phenanthrene d10 (31.61 mg l^−1^), anthracene d10 (28.56 mg l^−1^), fluoranthene d10 (27.59 mg l^−1^), pyrene d10 (27.78 mg l^−1^), chrysene d10 (2.12 mg l^−1^) and benzo[a]anthracene d12 (4.56 mg l^−1^, all obtained from Dr Ehrensdorfer GmbH, Augsburg, Germany). HgCl_2_ to a final concentration of 132 mg l^−1^ was added to prevent bacterial activity. The seawater samples were extracted with 1 ml of hexane (Lichrosolv, Merk Darmstadt Germany) for 6 h on a horizontal shaker, after which the extract was dried with precleaned, dry sodium sulfate. Acenaphthylene d08 was added as injection standard.

For nC_8_–nC_10_ alkane analysis, 10 ml of seawater sample was filled immediately after sampling into precleaned 20-ml bottles and directly afterwards spiked with 5 μl of a deuterated nonane-d20 standard mixture (91.2 mg l^−1^; Aldrich Chem. Co, USA, Milwaukee) and 2 μl of HgCl_2_ solution to reach a final concentration of 132 mg l^−1^. The seawater samples were then extracted with 1 ml of hexane (Lichrosolv, Merk Darmstadt Germany) for 6 h on a horizontal shaker, after which the extract was dried with precleaned, dry sodium sulfate, and acenaphthylene d08 was added as injection standard.

For analysis of benzene, toluene, ethylbenzene and xylenes, 10 ml of seawater sample was filled into precleaned 20-ml bottles and directly afterwards spiked with 2 μl of HgCl_2_ solution to reach a final concentration of 132 mg l^−1^. The concentration of benzene, toluene, ethylbenzene and xylenes was determined by headspace gas chromatography with flame ionization detector on a Hewlett Packard 6890 Series GC (Palo Alto, California, USA), using an automated headspace sampler (Hewlett Packard 7694), an oven temperature of 95 °C and an injection volume of 1 ml. Compounds were separated on a fused silica capillary column (Optima ∂-3, 60-m length, 0.25-mm internal diameter (i.d.), 0.35-μm film thickness; Macherey-Nagel, Düren, Germany), at a temperature sequence of 60 °C for 2 min, heat to 120 °C at a rate of 4 °C min^−1^, followed by a second gradient up to 280 °C at a rate of 20 °C min^−1^, and a subsequent cooling down to 60 °C. The flame ionization detector was operated at 280 °C and helium was used as a carrier gas under constant flow mode at 2.0 ml min^−1^.

PAHs were identified by gas chromatography with mass spectrum analysis using an HP 6890 Series GC, equipped with a 20-m HP5MS capillary column (0.18-mm i.d. and 0.18-μm film thickness) and 1-m uncoated and deactivated HP retention gap (0.18-mm i.d.). This was connected to an Agilent 5973 MSD (Palo Alto, California, USA) operating in selected ion monitoring mode. Splitless injection of 1-μl sample was performed using an automatic HP 7683 Series injector and an HP septumless programmed temperature vaporization (PTV) injector. The injector program was set at 80 °C for 0.02 min, followed by a ramp of 600 °C min^−1^ to 300 °C, which was held for 10 min. The oven temperature program was 50 °C for 2 min, followed by a ramp of 15 °C min^−1^ to 300 °C, which was held for 6.3 min. The analysis was run under constant flow mode at 1.0 ml min^−1^, with helium as the carrier gas.

Finally, *n*-alkanes were determined on the HP 6890 Series GC with a 60-m Optima delta-3 capillary column (0.25-mm i.d. and 0.25-μm film thickness, Macherey-Nagel), connected to the Agilent 5973 MSD operating in selected ion monitoring mode. Sample (0.5 μl) was splitless injected using the HP 7683 Series injector and septumless PTV injector. The injector program was 70 °C for 0.01 min, 600 °C min^−1^ to 250 °C, which was held for 10 min. The oven temperature program was 70 °C for 2 min, 10 °C min^−1^ to 180 °C and then 30 °C min^−1^ to 250 °C, which was held for 2 min. The analysis was run under constant flow mode at 1.0 ml min^−1^, with helium as the carrier gas.

Duplicate sample measurements sometimes varied substantially (for example, sample M5 in Supplementary Table 4), suggesting inclusion of small oil droplets in some seawater samples, which upon extraction would cause much higher apparent compound concentrations.

## Additional information

**How to cite this article:** Brussaard, C. P. D. *et al*. Immediate ecotoxicological effects of short-lived oil spills on marine biota. *Nat. Commun.* 7:11206 doi: 10.1038/ncomms11206 (2016).

## Supplementary Material

Supplementary Information Supplementary Figures 1-15, Supplementary Tables 1 - 6 and Supplementary References.

## Figures and Tables

**Table 1:** Estimated mass balance of the oil release and recovery for the experimental spills in 2008 and 2009.

**Compound classes**	**Site**							
	**17-6 /18-5 (2008)**				**M4 (2009)**			
	Total released mass* **(mg)**	**Measured concentration (μg l**^−1^**)**	**Total measured amount (mg)**	**Per cent detected**† **(%)**	Total released mass* **(mg)**	**Measured concentration (μg l**^−1^**)**	**Total measured amount (mg)**	**Per cent detected**† **(%)**
Total *n*-alkanes	3.7 × 10^8^	32.2‡	5.1 × 10^8^	141	4.0 × 10^7^	20.8‡	9.8 × 10^8^	2,460
Total BTEX	4.7 × 10^7^	ND	ND		6.0 × 10^6^	29.3‡	1.4 × 10^9^	22,900
Total PAH	3.4 × 10^7^	1.01‡	1.72 × 10^7^	50	2.8 × 10^7^	0.9‡	4.2 × 10^7^	151
C_6_–C_12_ *n*-alkanes	1.24 × 10^8^	ND	ND		3.1 × 10^6^	14.6‡	6.9 × 10^8^	21,900
Total Me-NAH/PHE	2.25 × 10^7^				2.27 × 10^7^			
OCT-equivalents§		0.04§	6.4 × 10^5^	0.5		0.2§	1.1 × 10^7^	342
TOL-equivalents§		1.7§	2.8 × 10^7^	59		0.8§	4.0 × 10^7^	634
NAH-equivalents§		1.0§	1.6 × 10^7^	73||		2.9§	1.4 × 10^8^	495||

BTEX, benzene, toluene, ethylbenzene and xylenes; NAH, naphthalene; ND, not determined; PHE, phenanthrenes; PAH, polycyclic aromatic hydrocarbon; TOL, toluene.

^*^Calculated from the oil composition, the released volume of oil (5 m^3^) and the estimated slick surface for location 17-6 (7 h after spill): 2 (km^2^), estimated corresponding sea volume below slick surface to a depth of 8 m: 1.6 × 10^7^ (m^3^). For location M4 (18 h after spill): 5.8 (km^2^) and 4.7 × 10^7^ (m^3^), respectively. For sites and locations, see Supplementary Table 6.

^†^As the total measured amount divided by the total released mass in %.

^‡^Measured using chemical analytics, averaged from 1.5- to 8-m depth (for ES-2008, 17-6 1.5-m depth and 18-5 8-m depth values).

^§^Measured using bacterial bioreporter analysis, averaged from 1.5- to 8-m depth if available. Concentrations converted to μg l^−1^ by assuming all available compound for the reporter cells being the standard used for calibration (that is, octane, toluene or naphthalene).

^||^On the basis of the total Me-NAH/PHE mass.
