# Supplementary material for: Immediate ecotoxicological effects of short-lived oil spills on marine biota
Source: Nat Commun. 2016 Apr 4;7:11206. doi: 10.1038/ncomms11206 (PMC4822028; doi:10.1038/ncomms11206)
Supplement: Supplementary Information — Supplementary Figures 1-15, Supplementary Tables 1 - 6 and Supplementary References. [file ncomms11206-s1.pdf]

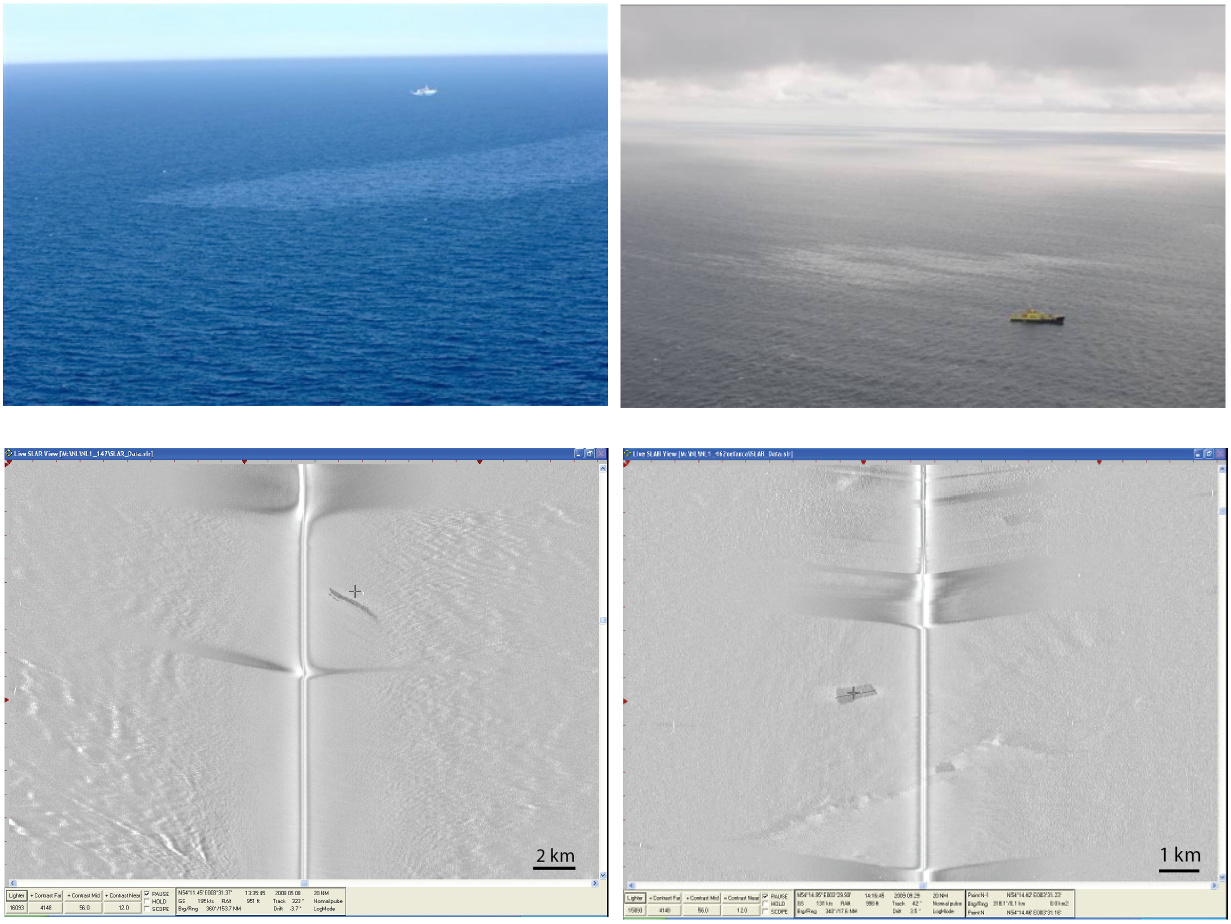

Supplementary Figure 1. Airplane observations from the ES-2008 and ES-2009 spills. Left panels, ES-2008 spill. Right panels, ES-2009 spill. Above, aerial photographs. Below; radar side scatter. +, position of the oil slick. Visible on left image: *RSS Pelagia*, on right image: *RSS Arca*.

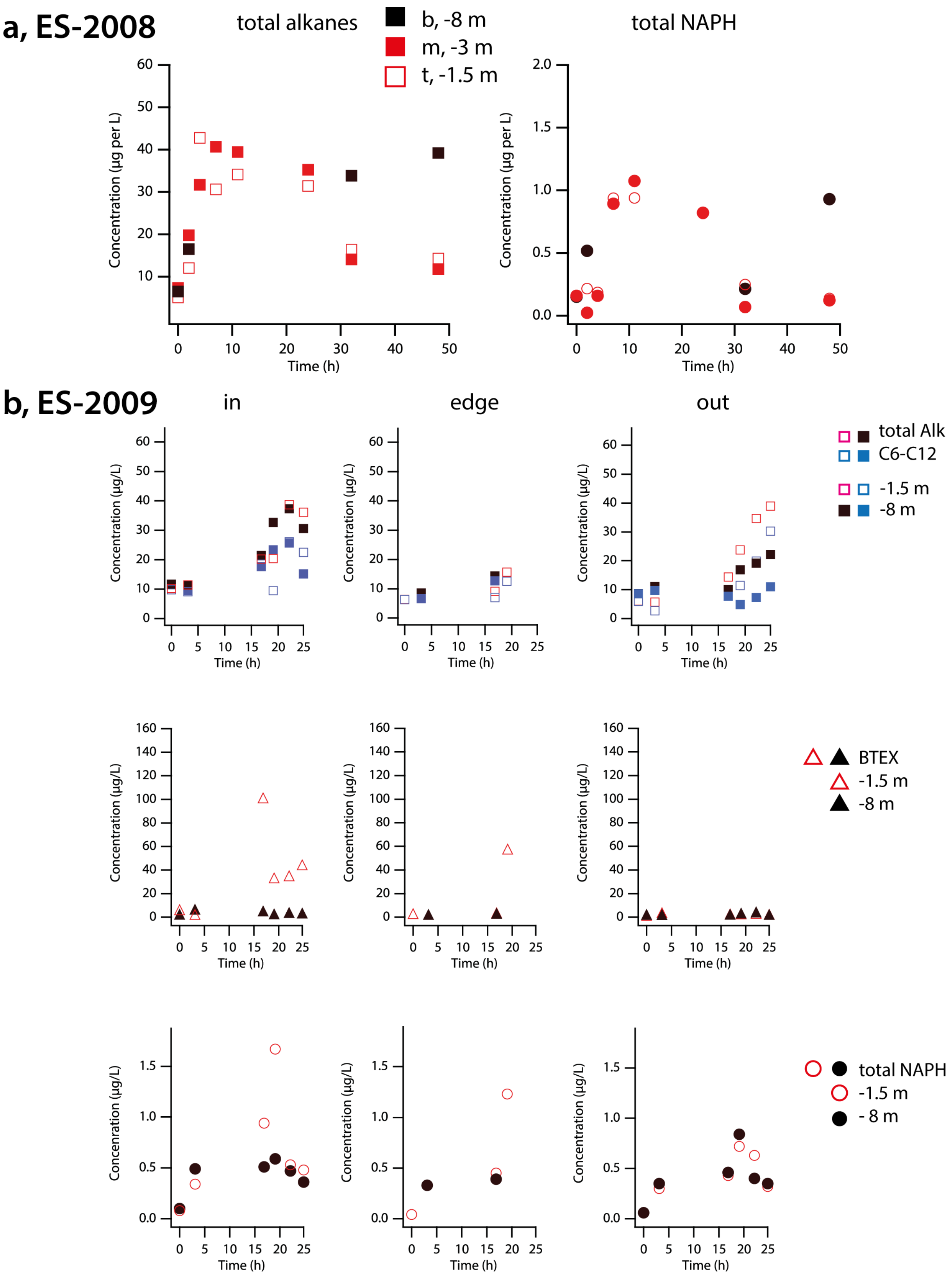

Supplementary Figure 2. Dissolution and weathering kinetics of dissolved oil components in the ES-2008 (a) and ES-2009 (b) spills, at all sampled depths. Total Naph, sum of naphthalene, methyl- and dimethylnaphthalenes. Total alk, sum of linear alkanes between C10 and C40 (ES-2008), or C6-C40 (ES-2009). BTEX, sum of benzene, toluene, ethylbenzene and xylenes.

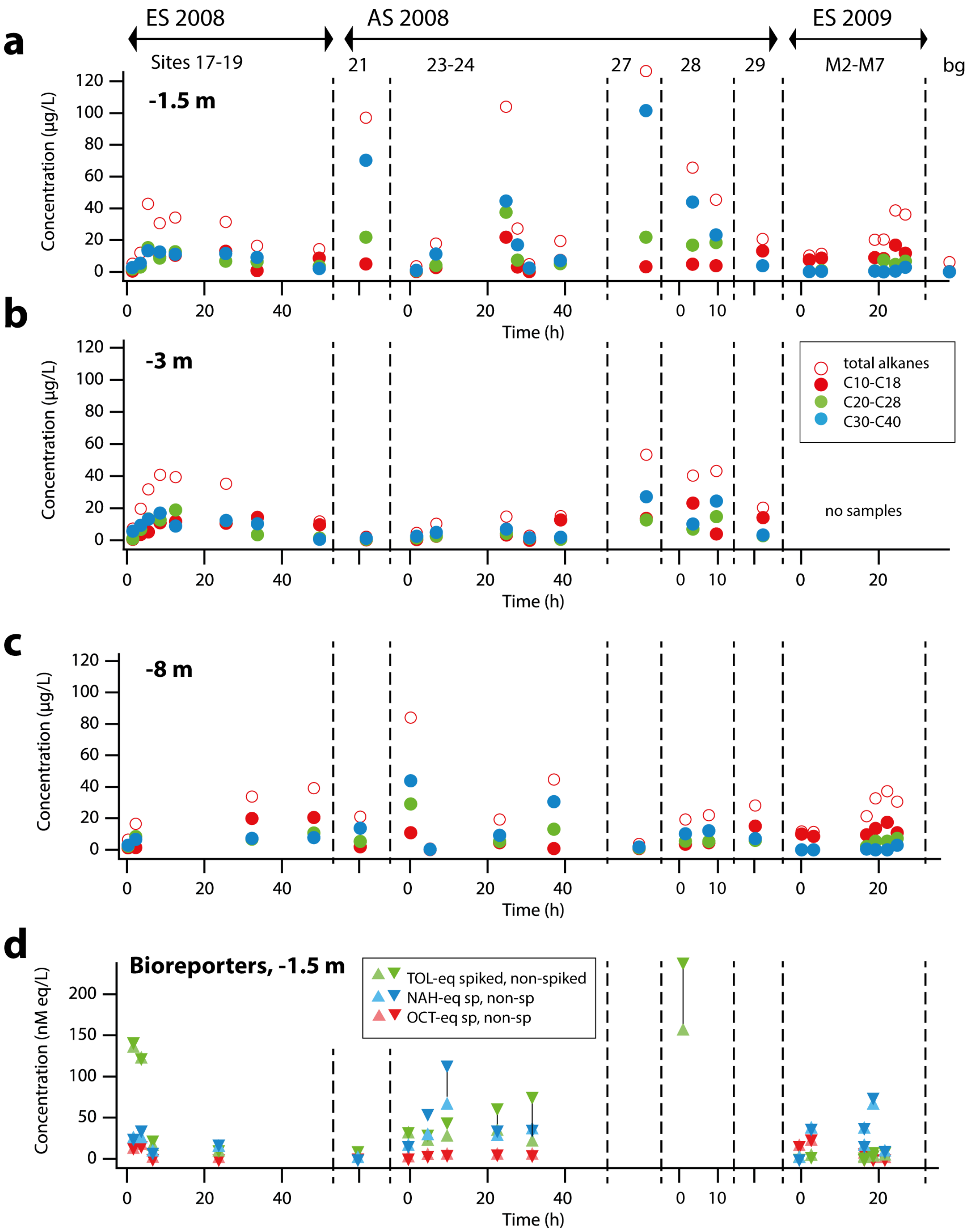

Supplementary Figure 3. Depth profile concentrations of sum alkane parameters and bioreporter measurements across all open sea spills. For description of sampling points and sites, see Table S2 and Fig. 1.

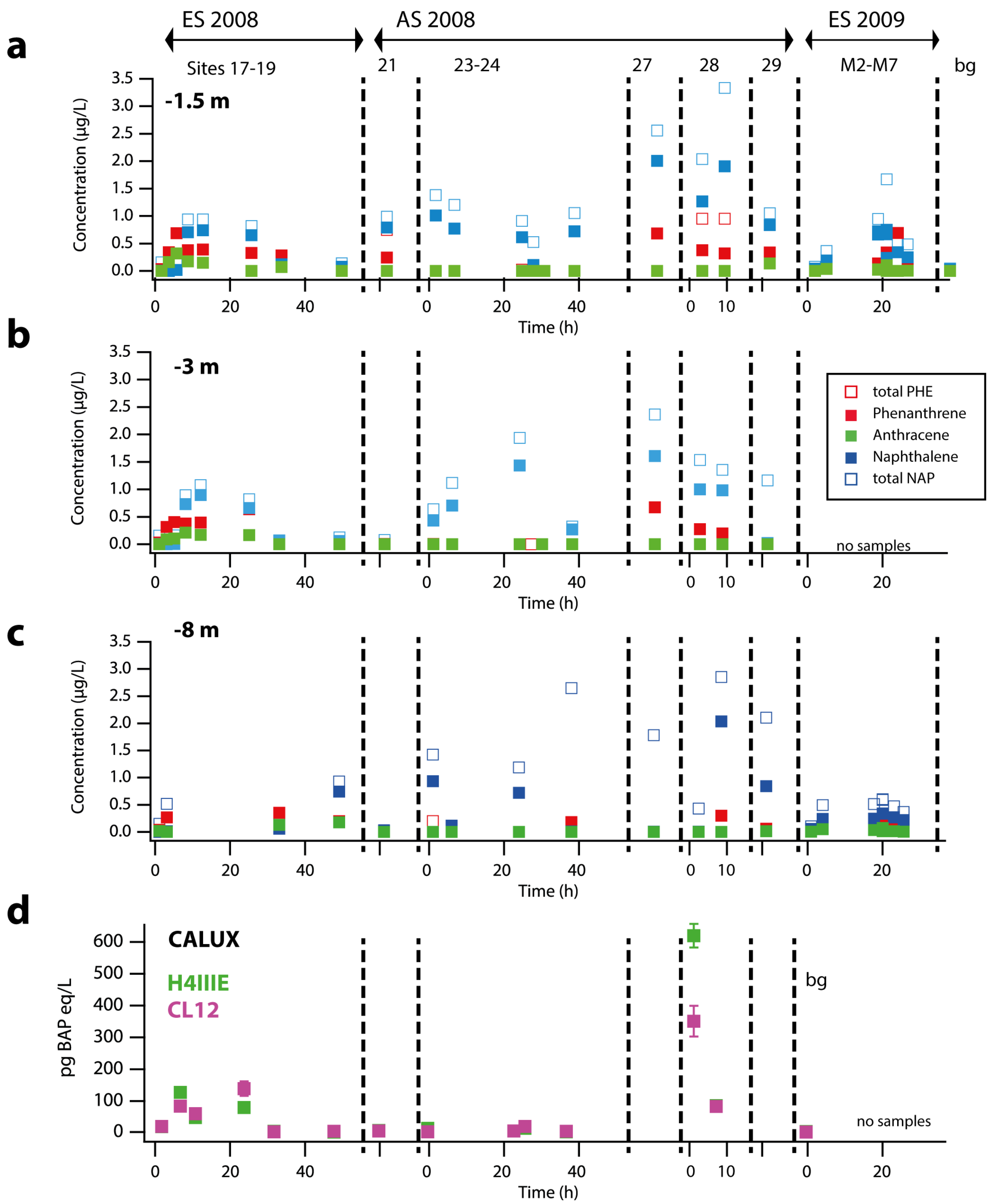

Supplementary Figure 4. Depth profiles of sum polycyclic aromatic hydrocarbons and CALUX toxicity tests across all open sea spills over time. For description of sampling points and sites, see Table S2 and Fig. 1.

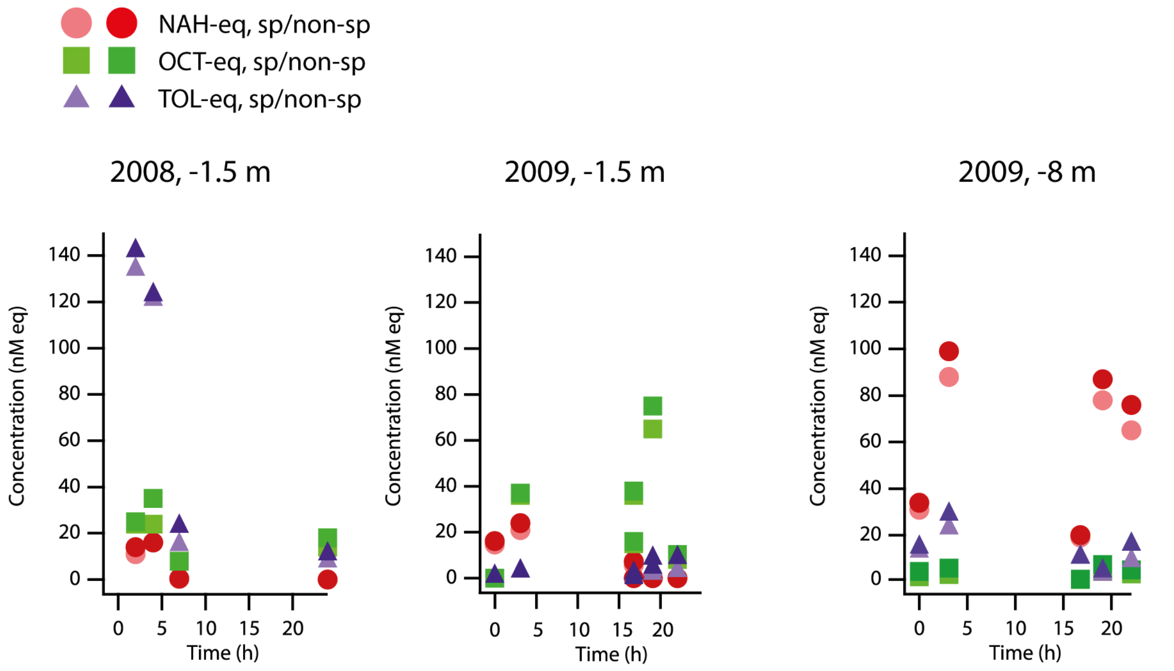

Supplementary Figure 5. Dissolution and weathering kinetics of the ES-2008 and ES-2009 spills analyzed on board with bioreporter assays. sp/non-sp, spiked /non-spiked samples.

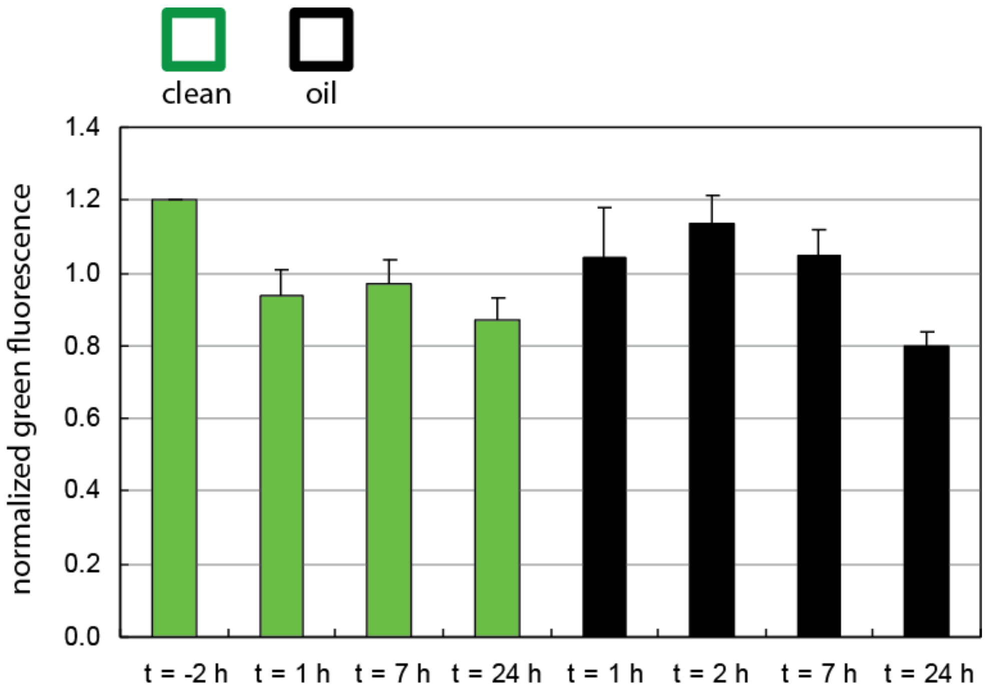

Supplementary Figure 6. *Micromonas pusilla* viability assay with samples derived from the ES-2008 spill (black bars) and with control samples from clean zones outside the spill (green bars). Values show normalized green fluorescence from cells exposed to seawater samples, compared to exposure in clean culture medium (=1). Bars are the average from the values of three depths at that sampling time point. Error bars indicate the difference between the minimum and maximum values. Time in h compared to spill release (t=0).

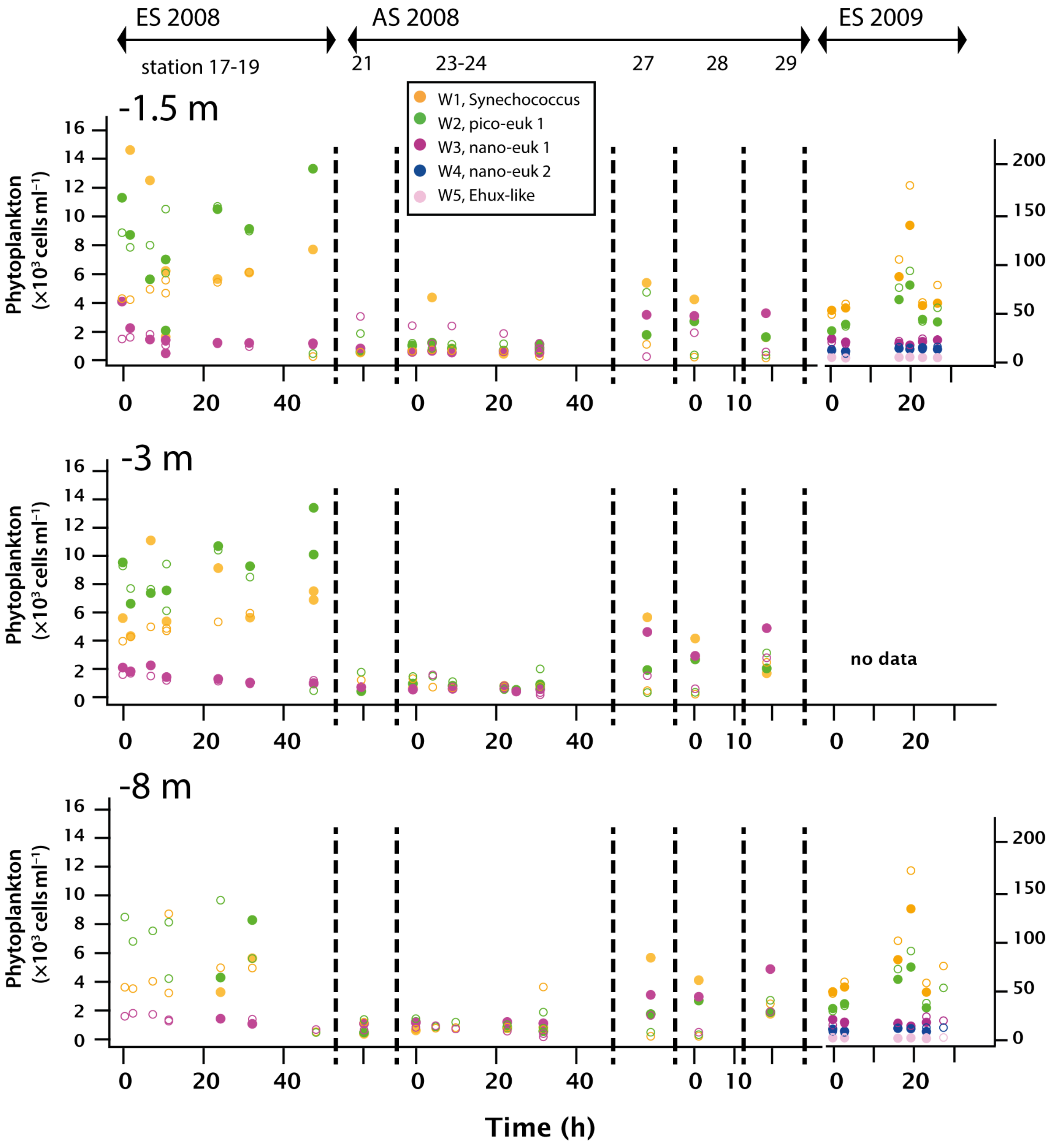

Supplementary Figure 7. Abundances of phytoplankton populations in seawater samples underneath (filled colors) and outside oil slicks (open circles), and at three different depths estimated by flow cytometry counting (1.5, 3 and 8 m below surface). Colors indicate different phytoplankton groups. ES-2008, experimental spill of 2008. AS 2008, encountered accidental spills in 2008. ES-2009, experimental spill of 2009. Note the different right-hand scale (y-axis) for counts in the ES-2009. For site description and locations, see Table S2 and Fig. 1.

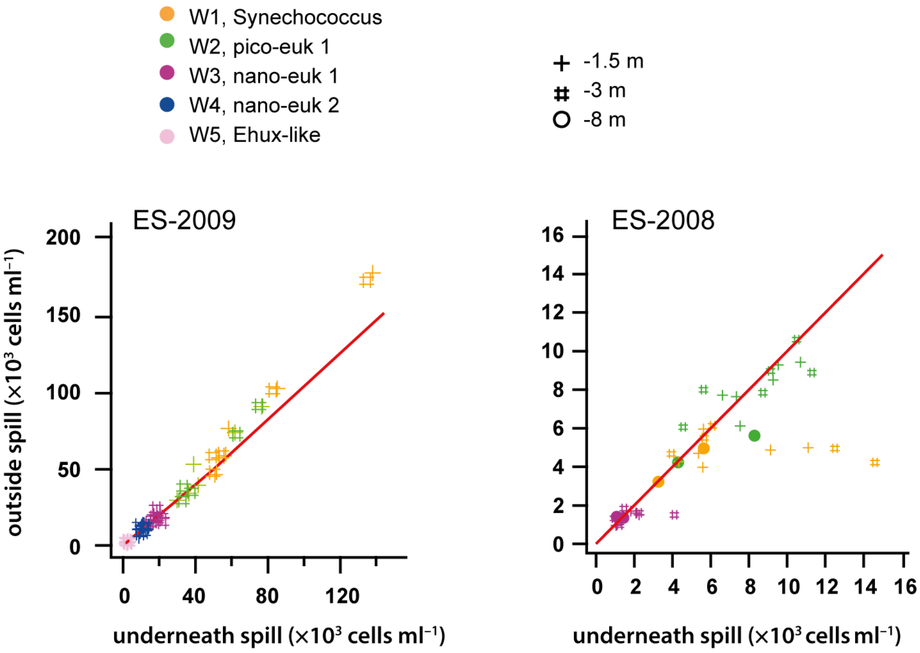

Supplementary Figure 8. Correlation between pair-wise plankton counts in seawater samples from below slicks with outside slicks sampled at similar time points. Different colors correspond to different phytoplankton groups. Different symbols correspond to different sampling depths. Note the difference in scale (phytoplankton abundance) between ES-2009 and ES-2008. The red line indicates the expected pair-wise abundance for identical counts below slicks and in clean seawater regions.

Abundances of total and picoeukaryotic phytoplankton in 2008 correlated well across all pair-wise (oil vs non-oil) samples, further supporting the interpretation that the oil did not affect total phytoplankton numbers (r^2 = 0.85-0.98).

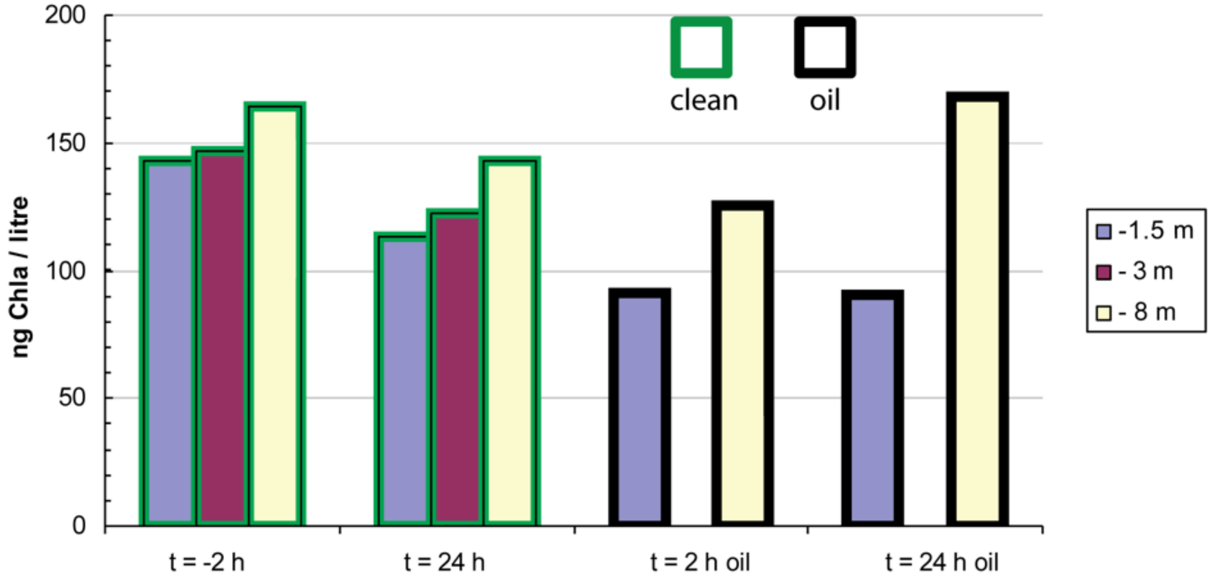

Supplementary Figure 9. Chlorophyl-a concentrations in samples from the ES-2008 spill (black outlines) and comparable outside-slick samples (green outlines), at three depths, as indicated. Values are not significantly different between oiled and non-oiled samples (t-test all oil/non-oil, two-tailed, unequal variance, P=0.27; t-test all samples at -1.5 m oil/non-oil, one-tailed, unequal variance, P=0.064).

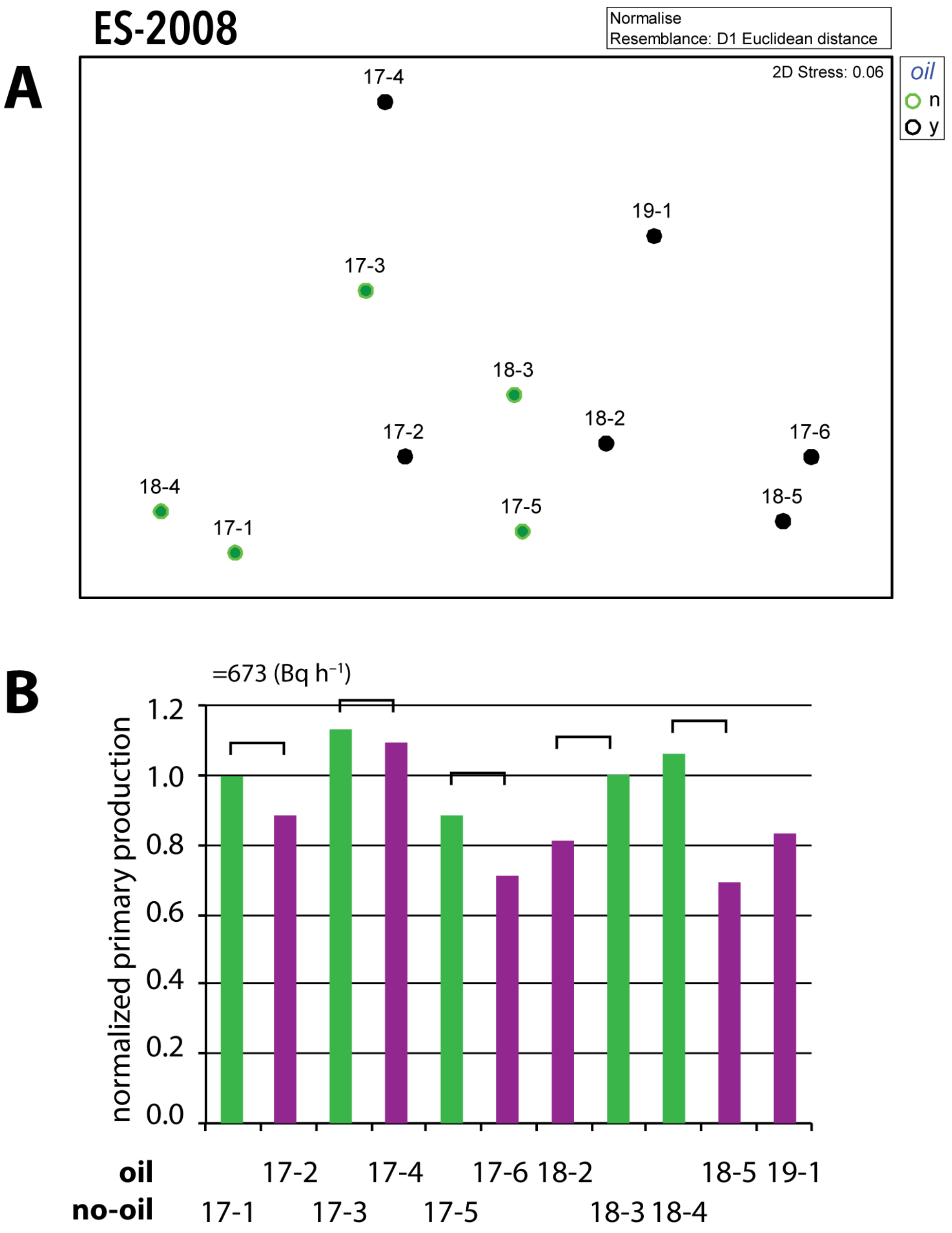

Supplementary Figure 10. Primary production in samples from the ES-2008 spill with oil (black) compared to outside-slick samples (green). (A) Multivariate analysis combining the four primary production curve variables *Pmax*, *Imax*, *s* and *w* (ANOSIM, Euclidian distance, $R = 0.26$, $P < 0.05$). (B) Normalized primary production compared to 17-1 (= 673 Bq h^{-1}). For sample and site indications, see Table S2. Samples taken at approximate similar time points grouped by clamps.

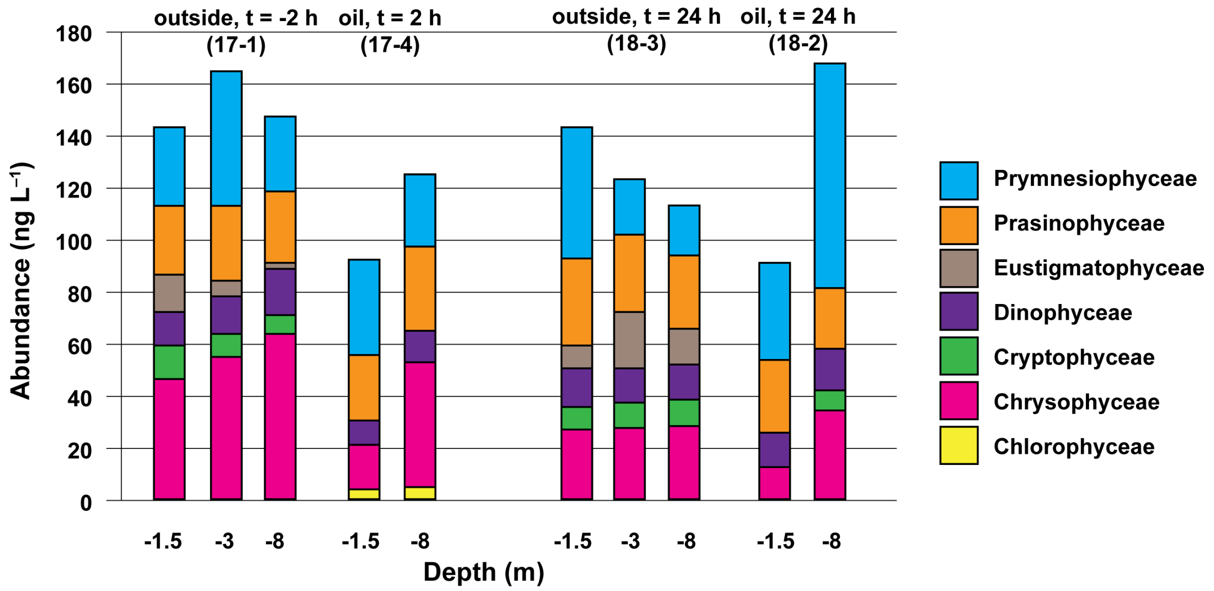

Supplementary Figure 11. Distribution of Phytoplankton families on the basis of photopigments in the ES-2008 spill among seawater samples from outside slick zones (sampling sites 17-1 and 18-3) versus underneath slick (17-4 and 18-2). Note the disappearance of Cryptophyceae and Eustigmatophyceae in oiled samples.

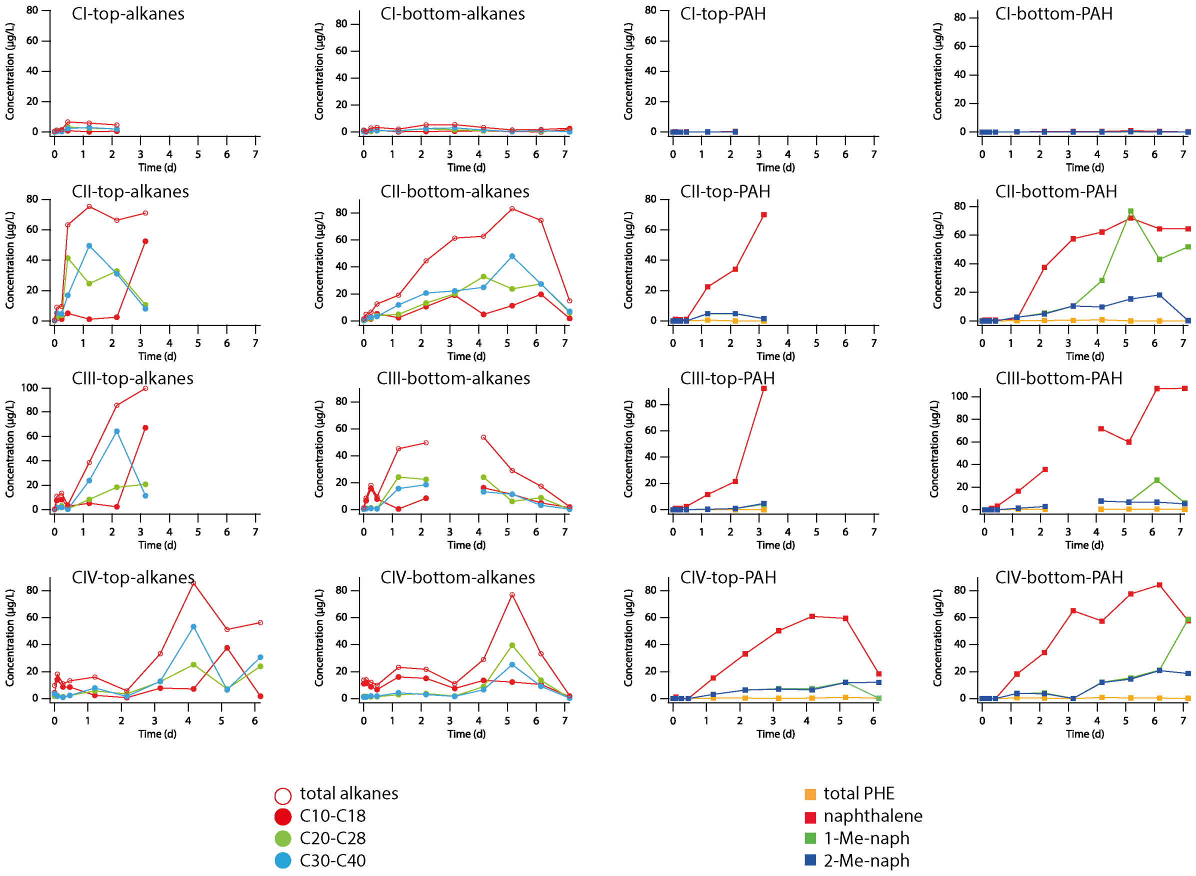

Supplementary Figure 12. Selected and sum parameter chemical data of mesocosm vessel samples over time.

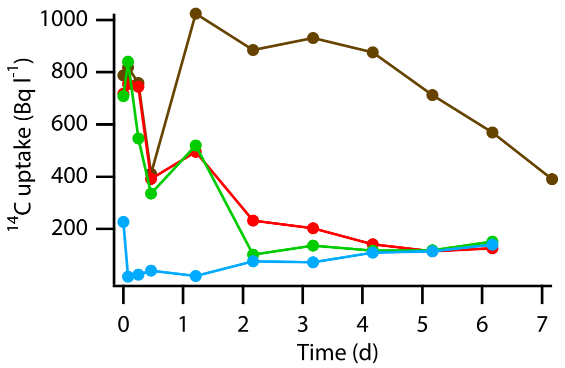

Supplementary Figure 13. ^{14}C-bicarbonate uptake within 2 h incubation at 72 µmol photons m^{-2} s^{-1} illumination for samples taken from the mesocosms at different time points. Brown, C-I (uncontaminated control); red, C-II (2 l crude oil); green, C-III (5 l crude oil); blue, C-IV (UV killed control but contaminated with 2 l crude oil).

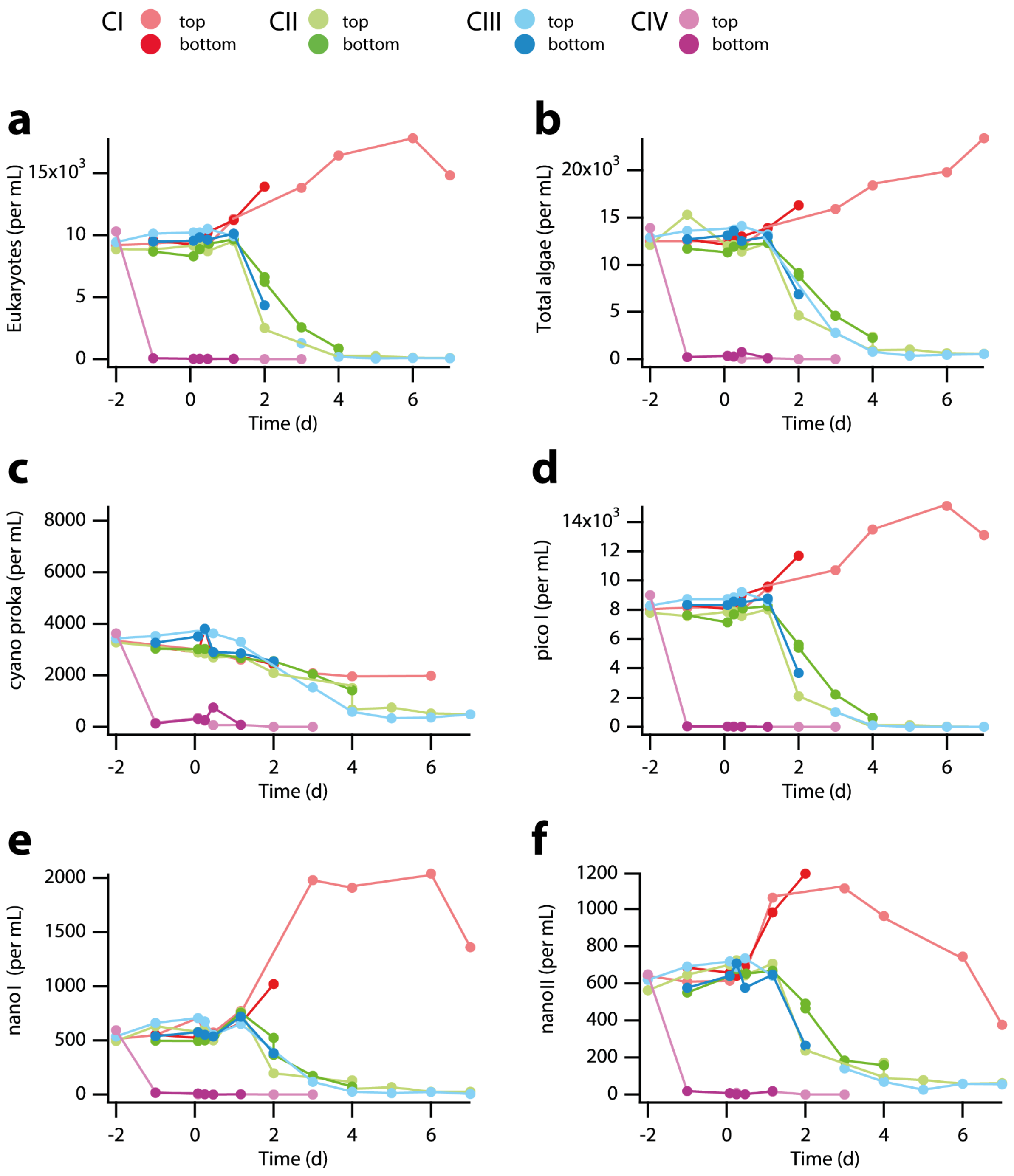

Supplementary Figure 14. Temporal phytoplankton abundance dynamics in the mesocosms. **a** Total phytoplankton counts. **b** Cyanobacteria (*Synechococcus* spp.). **c** Total eukaryotic phytoplankton. **d** Picoeukaryotic phytoplankton. **e** Nanoeukaryotic phytoplankton group I. **f** Nanoeukaryotic phytoplankton group II.

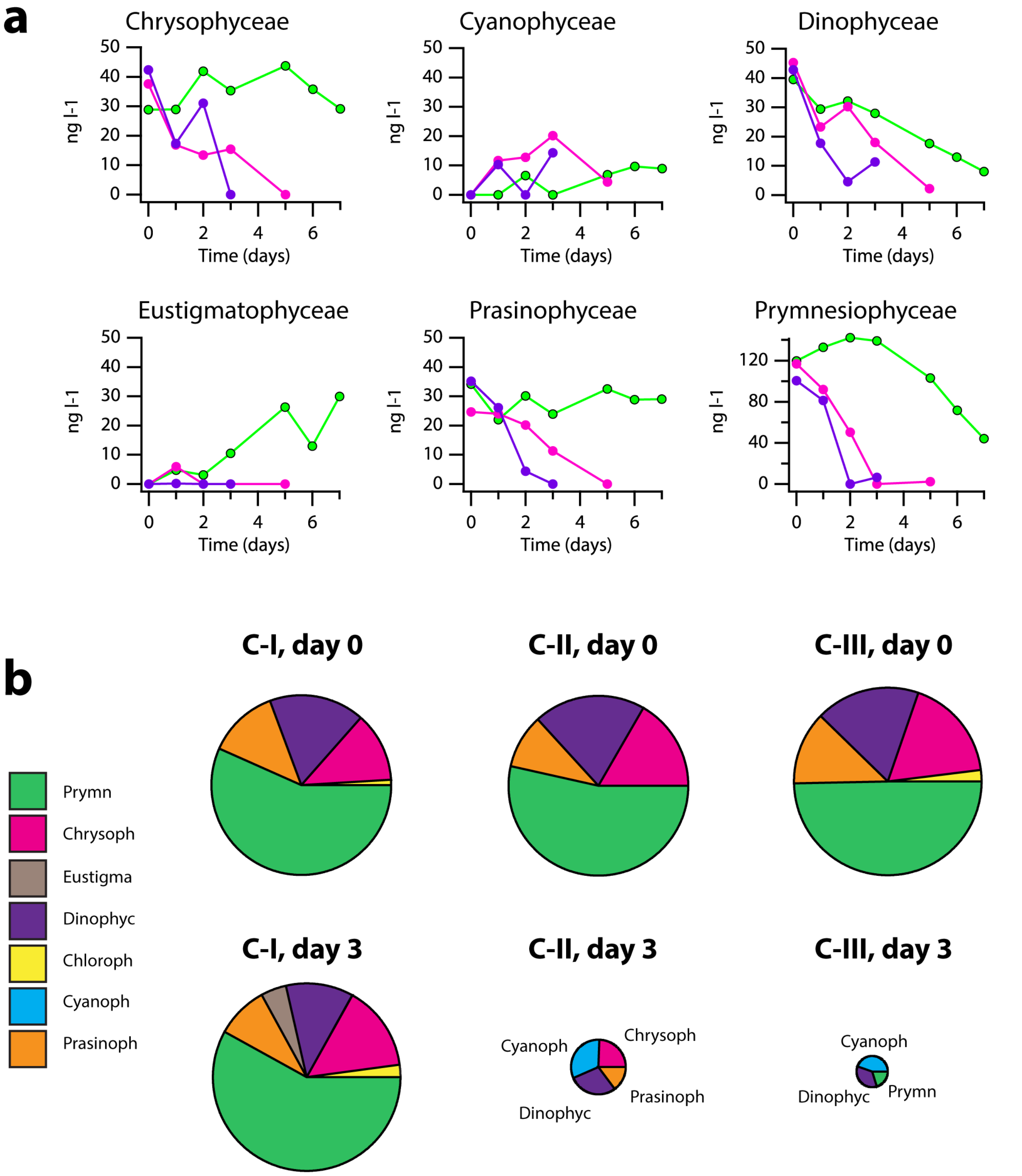

Supplementary Figure 15. Changes in major phytoplankton groups in samples from mesocosms determined by pigment analysis. **a** Changes over time in C-I (non-oiled, green), C-II (oiled, rose) and C-III (oiled, magenta). **b** Proportional composition at day 0 and day 3. Pie diameter proportional to total amount of pigment per ml sample. Note the disappearance of Chrysophyceae, Prymnesiophyceae and Prasinophyceae in oiled compared to non-oiled mesocosms.

Supplementary Table 1. Composition and basic properties of the crude oils used in the experimental spills.

**Property**[a]	**Arabian light (ES2008)**[b]	**Crane (ES2009)**	
API Gravity	33.4	19.10	° API
density	0.8641	0.963	g/ml
total BTEX	10.9	1.24	mg/g
total n-alkanes	84.6	8.26	mg/g
total C8-C11 alkanes	28.7	0.65	mg/g
naphthalene	50.3	832	μg/g
total methylated naphthalenes	3.9	4.9	mg/g
phenanthrene	51.2	338	μg/g
total methylated phenanthrenes	1.3	0.85	mg/g
total NAH,PHE,DBT,CHR,FLU	7.9	5.81	mg/g
total other PAHs	37	220	μg/g

a) at 0% weathering and 15°C.
b) Reference [1]
c) Reference [2]

Supplementary Table 2. Overview of sampling sites and measurement types.

Time (MET) and date	Site designation	Sample designation	Sampling time (h)	Position[a]	Depth (m)	Location of vessel	Measurements							
**ES 2008**														
	17-19	17-1	-2	O	1.5, 3, 8		Nu	Chl-A	Pl	PP	FDA	Chem		
08.05.08, 09:20		17-2	0	I	1.5, 3, 8	54°17’N, 3°50’E	Nu	Chl-A	Pl	PP	FDA	Chem	BB	
		17-3	1	O	1.5, 3, 8		Nu	Chl-A	Pl	PP	FDA			
11:20		17-4	2	I	1.5, 3, 8	54°17'N, 3°53'E	Nu	Chl-A	Pl	PP	FDA	Chem	BB	
		17-5	7	O	1.5, 3, 8		Nu	Chl-A	Pl	PP	FDA			
16:30		17-6	7	I	1.5, 3, 8	54°19'N, 3°52'E	Nu	Chl-A	Pl	PP	FDA	Chem	BB	CALUX
		17-7	11	O	1.5, 3, 8		Nu	Chl-A	Pl	PP	FDA			CALUX
20:00		17-8	11	I	1.5, 3, 8	54°20'N, 3°40'E	Nu	Chl-A	Pl	PP	FDA	Chem		
		18-1	23	O	1.5, 3, 8		Nu	Chl-A	Pl	PP	FDA			
09.05.2008, 09:30		18-2	24	I	1.5, 3, 8	54°22'N, 3°31'E	Nu	Chl-A	Pl	PP	FDA	Chem	BB	CALUX
		18-3	24	O	1.5, 3, 8		Nu	Chl-A	Pl	PP	FDA			
		18-4	31	O	1.5, 3, 8		Nu	Chl-A	Pl	PP	FDA			
17:00		18-5	32	I	1.5, 3, 8	54°25'N, 3°37'E	Nu	Chl-A	Pl	PP	FDA	Chem		CALUX
10.05.2008, 08:45		19-1	48	I	1.5, 3, 8	54°29'N, 3°32E	Nu	Chl-A	Pl	PP	FDA	Chem		CALUX
**ES 2009**														
29.09.09 14:10	M2-M7	M1	0	O	1.5, 8	54°09.822'N/3°30.308'E	Nu	Chl-A	Pl		FDA			
15:35		M2	1	O	1.5, 8	54°09.432'N/3°31.026'E	Nu	Chl-A	Pl		FDA	Chem		
			2	I, E	1.5, 8		Nu	Chl-A	Pl		FDA	Chem	BB	
18:42		M3	4	O	1.5, 8		Nu	Chl-A	Pl		FDA			
			5	I, E	1.5, 8		Nu	Chl-A	Pl		FDA	Chem	BB	
30.09.09 08:27		M4	18	O	1.5, 8	54°10.413'N/3°32.055'E	Nu	Chl-A	Pl		FDA			
			19	I, E	1.5, 8		Nu	Chl-A	Pl		FDA	Chem	BB	
10:41		M5	20	O	1.5, 8	54°08.861'N/3°29.967'E	Nu	Chl-A	Pl		FDA			
			21	I, E	1.5, 8		Nu	Chl-A	Pl		FDA	Chem	BB	
13:40		M6	23	O	1.5, 8	54°08.861'N/3°28.967'E	Nu	Chl-A	Pl		FDA			
			24	I	1.5, 8		Nu	Chl-A	Pl		FDA	Chem	BB	
16:20		M7	26	O, I	1.5, 8	54°08.803'N/3°31.393'E	Nu	Chl-A	Pl		FDA			
**AS 2008**														
10.05.08 20:05	21[b]	21-1	1*	I	1.5	53.83153 N, 3.97798 E	Nu	Chl-A	Pl	PP	FDA	Chem	BB	CALUX
11.05.08 10:00	23-24[c]	23-1	1*	O, I	1.5	53°45'N, 4°1'E	Nu	Chl-A	Pl	PP	FDA	Chem	BB	CALUX
		23-3	6	O, I	1.5		Nu	Chl-A	Pl	PP	FDA	Chem	BB	
		23-4	11	O, I	1.5		Nu	Chl-A	Pl	PP	FDA		BB	
		24-1	24	O, I	1.5	53.79067 N, 4.12058 E	Nu	Chl-A	Pl	PP	FDA	Chem	BB	CALUX
		24-2		O, I	1.5		Nu	Chl-A	Pl	PP	FDA	Chem		CALUX
		24-4	30	O, I	1.5		Nu	Chl-A	Pl	PP	FDA	Chem	BB	CALUX
14.05.08 08:00	**27**[d]	27-1	1*	O	1.5	53.50843 N, 3.96498 E	Nu	Chl-A	Pl	PP	FDA	Chem		
14.05.08 10:30	28[e]	28-1	1*	I	1.5	53.4065 N, 3.663 E	Nu	Chl-A	Pl	PP	FDA	Chem	BB	CALUX
		28-2	2.5	O	1.5	53.41993 N, 3.61137 E	Nu	Chl-A	Pl	PP	FDA	Chem		

14.05.08 15:30	**29**[d]	29-1	1*	O	1.5	53.57586 N, 3.89585 E	Nu	Chl-A	Pl	PP	FDA	Chem		
**Cube vessels**														
		CI-CIV	-2	b, t, m	na	na	Nu	Chl-A	Pl	PP	FDA	Chem		
07.05.08 09:45		CI-CIV	0	b, t, m	na		Nu	Chl-A	Pl	PP	FDA	Chem		
		CI-CIV	2	b, t, m	na		Nu	Chl-A	Pl	PP	FDA	Chem		CALUX
		CI-CIV	6	b, t, m	na		Nu	Chl-A	Pl	PP	FDA	Chem	BB	CALUX
		CI-CIV	12	b, t, m	na		Nu	Chl-A	Pl	PP	FDA	Chem		CALUX
		CI-CIV	24	b, t, m	na		Nu	Chl-A	Pl	PP	FDA	Chem	BB	CALUX
		CI-CIV	48	b, t, m	na		Nu	Chl-A	Pl	PP	FDA	Chem	BB	CALUX
		CI-CIV	72	b, t, m	na		Nu	Chl-A	Pl	PP	FDA	Chem	BB	CALUX
		CI-CIV	96	b, t, m	na		Nu	Chl-A	Pl	PP	FDA	Chem	BB	CALUX
		CI-CIV	120	b, m	na		Nu	Chl-A	Pl	PP	FDA	Chem	BB	CALUX
		CI-CIV	144	b, m	na		Nu	Chl-A	Pl	PP	FDA	Chem		CALUX
		CI-CIV	168	b, m	na		Nu	Chl-A	Pl	PP	FDA	Chem	BB	CALUX

*) relative timing compared to first sample taken at that site.
a) O, outside visible slick; I, inside; E, edge of slick.
b) Philippe Patch - (Length 1 mile / direction 065 degr.)
c) Cornelis Patch - (Length 4 miles / direction 030 degr.)
d) no visible oil slick
e) Flipje Patch - (length 300 m width 30 m / direction nno-ssw)

Supplementary Table 3. Hydrocarbon analysis of seawater samples in the ES-2008 and accidental spills of the 2008 campaign.

	Concentration (µg/L) in sample																		
**Compound**	**start**	**17-2 top**	**17-4 top**	**17-6 top**	**17-8 top**	**18-2 top**	**18-5 top**	**19-1 top**	**21-1 top**	**23-1 top**	**23-3 top**	**24-1 top**	**24-2 top**	**24-3 top**	**24-5 top**	**27-1 top**	**28-1 top**	**28-2 top**	**29-1 top**
**Depth -1.5 m**																			
Naphthalene	0.004	0.002	0.016	0.705	0.740	0.652	0.120	0.075	0.790	1.011	0.770	0.613	0.106	0.000	0.720	2.005	1.265	1.907	0.835
Acenaphthylene	0.000	0.000	0.000	0.153	0.000	0.000	0.000	0.000	0.000	0.000	0.000	0.000	0.000	0.000	0.000	0.000	0.000	0.000	0.000
Acenaphthene	0.000	0.000	0.000	0.000	0.082	0.023	0.013	0.000	0.001	0.000	0.000	0.000	0.000	0.000	0.000	0.000	0.000	0.000	0.001
Fluorene	0.016	0.000	0.000	0.178	0.000	0.000	0.000	0.000	0.000	0.000	0.000	0.000	0.000	0.000	0.000	0.000	0.000	0.000	0.000
Phenanthrene	0.032	0.340	0.684	0.375	0.391	0.326	0.281	0.000	0.243	0.000	0.000	0.000	0.000	0.000	0.000	0.683	0.375	0.321	0.338
Anthracene	0.001	0.163	0.321	0.174	0.146	0.000	0.073	0.000	0.001	0.000	0.000	0.000	0.000	0.000	0.000	0.000	0.000	0.000	0.135
Fluoranthene	0.000	0.000	0.000	0.000	0.000	0.000	0.000	0.000	0.000	0.000	0.000	0.000	0.000	0.000	0.000	0.000	0.000	0.000	0.000
Pyrene	0.000	0.000	0.000	0.000	0.000	0.000	0.000	0.000	0.000	0.000	0.000	0.000	0.000	0.000	0.000	0.000	0.000	0.000	0.000
Chrysene	0.000	0.000	0.000	0.000	0.000	0.000	0.000	0.000	0.000	0.000	0.000	0.000	0.000	0.000	0.000	0.000	0.000	0.000	0.000
Benzo(a)anthracene	0.000	0.000	0.000	0.000	0.000	0.000	0.000	0.000	0.000	0.000	0.000	0.000	0.000	0.000	0.000	0.000	0.000	0.000	0.000
Benzo(b)fluoranthene	0.000	0.000	0.000	0.000	0.000	0.000	0.000	0.000	0.000	0.000	0.000	0.000	0.000	0.000	0.000	0.000	0.000	0.000	0.000
Benzo(k)fluoranthene	0.000	0.000	0.000	0.000	0.000	0.000	0.000	0.000	0.000	0.000	0.000	0.000	0.000	0.000	0.000	0.000	0.000	0.000	0.000
Benzo(a)pyrene	0.000	0.000	0.000	0.000	0.000	0.000	0.000	0.000	0.000	0.000	0.000	0.000	0.000	0.000	0.000	0.000	0.000	0.000	0.000
Indeno(123-cd)pyrene	0.000	0.000	0.000	0.000	0.000	0.000	0.000	0.000	0.000	0.000	0.000	0.000	0.000	0.000	0.000	0.000	0.000	0.000	0.000
Dibenzo(ah)anthracene	0.000	0.000	0.000	0.000	0.000	0.000	0.000	0.000	0.000	0.000	0.000	0.000	0.000	0.000	0.000	0.000	0.000	0.000	0.000
Benzo(ghi)perylen	0.000	0.000	0.000	0.000	0.000	0.000	0.000	0.000	0.000	0.000	0.000	0.000	0.000	0.000	0.000	0.000	0.000	0.000	0.000
2 Methylnaphthalene	0.070	0.107	0.085	0.111	0.101	0.070	0.070	0.023	0.097	0.132	0.149	0.126	0.145	0.001	0.104	0.182	0.285	0.584	0.104
1 Methylnaphthalene	0.077	0.107	0.085	0.122	0.099	0.093	0.058	0.025	0.098	0.188	0.168	0.158	0.143	0.001	0.115	0.225	0.298	0.480	0.109
2,6-Dimethylnaphthalene	0.003	0.000	0.000	0.000	0.000	0.000	0.000	0.005	0.000	0.051	0.039	0.013	0.047	0.000	0.034	0.050	0.073	0.150	0.000
1,6- Dimethylnaphthalene	0.004	0.000	0.000	0.000	0.000	0.000	0.000	0.011	0.000	0.000	0.079	0.000	0.086	0.000	0.081	0.095	0.117	0.212	0.000
1,2- Dimethylnaphthalene	0.000	0.000	0.000	0.000	0.000	0.000	0.000	0.000	0.000	0.000	0.000	0.000	0.000	0.000	0.000	0.000	0.000	0.000	0.000
3- Methylanthracene	0.000	0.000	0.000	0.000	0.000	0.000	0.000	0.000	0.000	0.000	0.000	0.000	0.000	0.000	0.000	0.000	0.000	0.000	0.000
3 -Methylphenanthrene	0.000	0.000	0.000	0.000	0.000	0.000	0.000	0.000	0.000	0.000	0.000	0.000	0.000	0.000	0.000	0.000	0.000	0.000	0.000
2 -Methylphenanthrene	0.000	0.000	0.000	0.000	0.000	0.000	0.000	0.000	0.000	0.000	0.000	0.000	0.000	0.000	0.000	0.000	0.000	0.000	0.000
9 -Methylphenanthrene	0.000	0.000	0.000	0.000	0.000	0.000	0.000	0.000	0.000	0.000	0.000	0.000	0.000	0.000	0.000	0.000	0.000	0.000	0.000
1 -Methylphenanthrene	0.000	0.000	0.000	0.000	0.000	0.000	0.000	0.000	0.000	0.000	0.000	0.000	0.000	0.000	0.000	0.000	0.000	0.000	0.000
1,7- Dimethylphenanthrene	0.000	0.000	0.000	0.000	0.000	0.000	0.000	0.000	0.502	0.000	0.000	0.020	0.000	0.000	0.000	0.000	0.575	0.629	0.000
1+3 -Methylfluoranthene	0.000	0.000	0.000	0.000	0.000	0.000	0.000	0.000	0.000	0.000	0.000	0.000	0.000	0.000	0.000	0.000	0.000	0.000	0.000
Retene	0.000	0.000	0.000	0.000	0.000	0.000	0.000	0.000	0.000	0.000	0.000	0.000	0.000	0.000	0.000	0.000	0.000	0.000	0.000
3 -Methylpyrene	0.000	0.000	0.000	0.000	0.000	0.000	0.000	0.000	0.000	0.000	0.000	0.000	0.000	0.000	0.000	0.000	0.000	0.000	0.000
1 -Methylpyrene	0.000	0.000	0.000	0.000	0.000	0.000	0.000	0.000	0.000	0.000	0.000	0.000	0.000	0.000	0.000	0.000	0.000	0.000	0.000
3 -Methylchrysene	0.000	0.000	0.000	0.000	0.000	0.000	0.000	0.000	0.000	0.000	0.000	0.000	0.000	0.000	0.000	0.000	0.000	0.000	0.000
6 -Methylchrysene	0.000	0.000	0.000	0.000	0.000	0.000	0.000	0.000	0.000	0.000	0.000	0.000	0.000	0.000	0.000	0.000	0.000	0.000	0.000
										ohne filter									
n-Decane	0.026	0.082	2.150	1.227	0.274	0.368	0.089	5.894	0.649	0.002	0.000	0.199	0.008	0.000	2.277	2.462	0.000	0.000	0.486
n-Dodecane	0.060	1.621	8.424	2.637	2.888	2.990	0.332	0.004	0.764	0.036	0.784	2.018	0.472	0.018	1.588	0.157	0.652	0.572	2.738
n-Tetradecane	0.042	1.193	2.959	3.134	3.929	7.841	0.239	2.500	1.781	0.075	1.941	18.689	2.527	0.060	3.392	0.470	3.712	2.811	6.517

n-Hexadecane	0.196	0.337	0.355	0.943	1.124	0.087	0.027	0.250	0.186	0.000	0.000	0.957	0.004	0.013	0.016	0.043	0.509	0.433	0.164
n-Heptadecane	0.185	0.117	0.104	0.504	0.742	0.675	0.031	0.000	0.011	0.002	0.000	0.000	0.000	0.000	0.000	0.000	0.000	0.000	1.250
Pristane	0.025	0.119	0.125	0.495	0.702	0.677	0.020	0.000	0.003	0.002	0.000	0.000	0.000	0.000	0.000	0.000	0.000	0.000	1.366
n-Octadecane	0.026	0.120	0.193	0.525	0.691	0.427	0.044	0.000	1.606	0.002	0.000	0.000	0.000	0.000	0.000	0.000	0.000	0.000	0.576
Phytane	0.026	0.125	0.177	0.244	0.652	0.428	0.025	0.000	0.011	0.002	0.000	0.206	0.000	0.000	0.000	0.000	0.000	0.000	0.580
n-Eicosane	0.249	0.206	0.360	0.235	0.256	0.206	0.102	0.000	0.004	0.001	0.000	0.201	0.003	0.009	0.006	0.000	0.000	0.320	0.374
n-Docosane	0.077	0.079	1.487	0.343	0.831	0.459	0.253	0.163	0.218	0.007	0.015	1.078	0.051	0.050	0.064	0.010	0.403	1.158	0.160
n-Tetracosane	0.664	0.287	4.010	1.618	5.311	1.236	1.138	0.905	0.844	0.084	0.471	6.449	0.961	0.353	0.999	2.177	1.763	3.240	0.687
n-Hexacosane	0.356	0.593	3.794	2.313	2.303	1.340	1.677	1.276	3.659	0.203	0.867	14.098	2.120	0.782	1.726	5.687	4.030	5.122	0.843
n-Octacosane	0.459	1.679	5.385	3.842	3.383	3.106	3.193	1.128	17.072	0.283	2.588	15.449	4.222	1.038	2.356	13.909	10.572	8.608	1.166
n-Triacontane	0.757	2.492	6.002	4.827	3.922	4.614	4.022	0.966	29.877	0.335	4.078	18.396	6.193	1.073	2.487	22.979	16.752	10.185	1.215
n-Dotriacontane	0.680	1.342	3.050	3.048	2.491	2.571	2.129	0.653	16.829	0.301	2.524	15.532	4.164	0.618	1.971	19.468	10.669	5.278	0.716
n-Tetratricontane	0.538	0.826	1.990	1.980	2.013	1.669	1.397	0.374	10.151	0.115	1.837	3.402	2.827	0.408	0.883	14.482	6.725	3.390	0.513
n-Hexatriacontane	0.447	0.612	1.400	1.715	1.770	1.945	1.077	0.148	8.162	0.146	1.743	3.484	2.283	0.195	1.525	44.538	5.805	2.526	0.778
n-Octatriacontane	0.284	0.193	0.811	1.008	0.829	0.748	0.579	0.002	5.239	0.068	1.007	3.734	1.471	0.123	0.170	0.026	4.078	1.819	0.558
n-Tetracontane	3.711	10.153	14.182	29.156	9.095	11.248	7.960	0.000	1.431	1.855	2.096	9.719	31.741	0.068	0.094	0.000	122.964	50.839	26.755
**Depth -3 m**																			
**Compound**	**start**	**17-2 mid**	**17-4 mid**	**17-6 mid**	**17-8 mid**	**18-2 mid**	**18-5 mid**	**19-1 mid**	**21-1 mid**	**23-1 mid**	**23-3 mid**	**24-1 mid**	**24-2 mid**	**24-3 mid**	**24-5 mid**	**27-1 mid**	**28-1 mid**	**28-2 mid**	**29-1 mid**
Naphthalene	0.003	0.002	0.003	0.730	0.896	0.661	0.065	0.058	0.000	0.432	0.704	1.436	-	0.000	0.268	1.607	0.999	0.984	0.023
Acenaphthylene	0.000	0.000	0.000	0.098	0.000	0.000	0.000	0.000	0.000	0.000	0.000	0.000	-	0.000	0.000	0.000	0.000	0.000	0.000
Acenaphthene	0.000	0.000	0.000	0.000	0.022	0.002	0.000	0.000	0.000	0.000	0.000	0.000	-	0.000	0.000	0.000	0.000	0.000	0.000
Fluorene	0.000	0.083	0.000	0.000	0.000	0.000	0.046	0.000	0.002	0.000	0.000	0.000	-	0.000	0.000	0.000	0.000	0.000	0.000
Phenanthrene	0.033	0.313	0.403	0.377	0.396	0.639	0.002	0.000	0.005	0.003	0.000	0.000	-	0.000	0.000	0.672	0.273	0.195	0.004
Anthracene	0.001	0.088	0.103	0.212	0.172	0.164	0.001	0.000	0.000	0.000	0.000	0.000	-	0.000	0.000	0.000	0.000	0.000	0.001
Fluoranthene	0.000	0.000	0.000	0.000	0.000	0.000	0.000	0.000	0.000	0.000	0.000	0.000	-	0.000	0.000	0.000	0.000	0.000	0.000
Pyrene	0.000	0.000	0.000	0.000	0.000	0.000	0.000	0.000	0.000	0.000	0.000	0.000	-	0.000	0.000	0.000	0.000	0.000	0.000
Chrysene	0.000	0.000	0.000	0.000	0.000	0.000	0.000	0.000	0.000	0.000	0.000	0.000	-	0.000	0.000	0.000	0.000	0.000	0.000
Benzo(a)anthracene	0.000	0.000	0.000	0.000	0.000	0.000	0.000	0.000	0.000	0.000	0.000	0.000	-	0.000	0.000	0.000	0.000	0.000	0.000
Benzo(b)fluoranthene	0.000	0.000	0.000	0.000	0.000	0.000	0.000	0.000	0.000	0.000	0.000	0.000	-	0.000	0.000	0.000	0.000	0.000	0.000
Benzo(k)fluoranthene	0.000	0.000	0.000	0.000	0.000	0.000	0.000	0.000	0.000	0.000	0.000	0.000	-	0.000	0.000	0.000	0.000	0.000	0.000
Benzo(a)pyrene	0.000	0.000	0.000	0.000	0.000	0.000	0.000	0.000	0.000	0.000	0.000	0.000	-	0.000	0.000	0.000	0.000	0.000	0.000
Indeno(123-cd)pyrene	0.000	0.000	0.000	0.000	0.000	0.000	0.000	0.000	0.000	0.000	0.000	0.000	-	0.000	0.000	0.000	0.000	0.000	0.000
Dibenzo(ah)anthracene	0.000	0.000	0.000	0.000	0.000	0.000	0.000	0.000	0.000	0.000	0.000	0.000	-	0.000	0.000	0.000	0.000	0.000	0.000
Benzo(ghi)perylen	0.000	0.000	0.000	0.000	0.000	0.000	0.000	0.000	0.000	0.000	0.000	0.000	-	0.000	0.000	0.000	0.000	0.000	0.000
													-						
2 Methylnaphthalene	0.076	0.011	0.078	0.063	0.091	0.073	0.002	0.024	0.010	0.081	0.152	0.172	-	0.000	0.029	0.240	0.201	0.123	0.557
1 Methylnaphthalene	0.080	0.010	0.078	0.100	0.088	0.086	0.002	0.021	0.014	0.100	0.144	0.188	-	0.001	0.027	0.291	0.180	0.123	0.583
2,6, Dimethylnaphthalene	0.000	0.000	0.000	0.000	0.000	0.000	0.000	0.005	0.009	0.023	0.042	0.048	-	0.000	0.000	0.056	0.056	0.037	0.000
1,6, Dimethylnaphthalene	0.000	0.000	0.000	0.000	0.000	0.000	0.000	0.016	0.041	0.000	0.077	0.096	-	0.000	0.000	0.172	0.099	0.088	0.000
1,2, Dimethylnaphthalene	0.000	0.000	0.000	0.000	0.000	0.000	0.000	0.000	0.000	0.000	0.000	0.000	-	0.000	0.000	0.000	0.000	0.000	0.000
3 Methylanthracene	0.000	0.000	0.000	0.000	0.000	0.000	0.000	0.000	0.000	0.000	0.000	0.000	-	0.000	0.000	0.000	0.000	0.000	0.000
3 Methylphenanthrene	0.000	0.000	0.000	0.000	0.000	0.000	0.000	0.000	0.000	0.000	0.000	0.000	-	0.000	0.000	0.000	0.000	0.000	0.000

2 Methylphenanthrene	0.000	0.000	0.000	0.000	0.000	0.000	0.000	0.000	0.000	0.000	0.000	0.000	-	0.000	0.000	0.000	0.000	0.000	0.000
9 Methylphenanthrene	0.000	0.000	0.000	0.000	0.000	0.000	0.000	0.000	0.000	0.000	0.000	0.000	-	0.000	0.000	0.000	0.000	0.000	0.000
1 Methylphenanthrene	0.000	0.000	0.000	0.000	0.000	0.000	0.000	0.000	0.000	0.000	0.000	0.000	-	0.000	0.000	0.000	0.000	0.000	0.000
1,7 Dimethylphenanthrene	0.000	0.000	0.000	0.000	0.000	0.000	0.000	0.000	0.000	0.000	0.000	0.000	-	0.000	0.000	0.000	0.000	0.000	0.000
1+3 Methylfluoranthene	0.000	0.000	0.000	0.000	0.000	0.000	0.000	0.000	0.000	0.000	0.000	0.000	-	0.000	0.000	0.000	0.000	0.000	0.000
Retene	0.000	0.000	0.000	0.000	0.000	0.000	0.000	0.000	0.000	0.000	0.000	0.000	-	0.000	0.000	0.000	0.000	0.000	0.000
3 Methylpyrene	0.000	0.000	0.000	0.000	0.000	0.000	0.000	0.000	0.000	0.000	0.000	0.000	-	0.000	0.000	0.000	0.000	0.000	0.000
1 Methylpyrene	0.000	0.000	0.000	0.000	0.000	0.000	0.000	0.000	0.000	0.000	0.000	0.000	-	0.000	0.000	0.000	0.000	0.000	0.000
3 Methylchrysene	0.000	0.000	0.000	0.000	0.000	0.000	0.000	0.000	0.000	0.000	0.000	0.000	-	0.000	0.000	0.000	0.000	0.000	0.000
6 Methylchrysene	0.000	0.000	0.000	0.000	0.000	0.000	0.000	0.000	0.000	0.000	0.000	0.000	-	0.000	0.000	0.000	0.000	0.000	0.000
													-						
n-Decane	0.008	0.129	0.185	1.881	1.824	0.356	0.040	6.147	0.006	0.009	0.000	0.000	-	0.000	4.724	3.654	0.000	0.000	0.501
n-Dodecane	0.025	2.027	1.641	2.819	2.961	3.327	0.077	0.737	0.010	0.174	0.445	0.401	-	0.016	7.768	2.548	0.586	0.513	3.020
n-Tetradecane	0.011	0.365	1.834	3.777	3.850	4.965	0.153	2.750	0.252	0.324	2.115	2.765	-	0.043	0.124	6.895	22.222	3.040	7.388
n-Hexadecane	0.321	0.539	0.446	0.990	1.205	0.953	0.022	0.017	0.047	0.008	0.000	0.212	-	0.008	0.002	0.506	0.394	0.425	0.407
n-Heptadecane	0.115	0.180	0.389	0.481	0.798	0.379	0.006	0.000	0.000	0.004	0.000	0.000	-	0.000	0.000	0.000	0.000	0.000	1.107
Pristane	0.002	0.178	0.355	0.512	0.826	0.321	0.006	0.000	0.000	0.009	0.000	0.000	-	0.000	0.000	0.000	0.000	0.000	1.153
n-Octadecane	0.115	0.185	0.350	0.551	0.201	0.293	0.006	0.000	0.002	0.018	0.000	0.003	-	0.000	0.002	0.000	0.000	0.000	0.497
Phytane	0.003	0.192	0.153	0.268	0.197	0.345	0.008	0.000	0.000	0.022	0.000	0.003	-	0.000	0.000	0.000	0.000	0.000	0.492
n-Eicosane	0.119	0.173	0.208	0.269	0.511	0.566	0.008	0.000	0.044	0.005	0.000	0.007	-	0.000	0.001	0.000	0.003	0.004	0.143
n-Docosane	0.086	0.488	0.556	0.794	0.588	0.800	0.017	0.014	0.049	0.043	0.027	0.052	-	0.021	0.013	0.911	0.203	0.681	0.171
n-Tetracosane	0.215	1.274	4.952	2.631	8.308	2.948	0.210	0.579	0.247	0.184	0.520	0.818	-	0.141	0.102	2.304	1.278	2.430	0.629
n-Hexacosane	0.293	1.603	3.490	3.338	4.809	3.178	0.666	0.366	0.170	0.377	0.946	1.333	-	0.360	0.270	3.397	2.260	4.151	0.477
n-Octacosane	0.270	3.060	3.983	5.368	4.441	4.394	2.574	0.420	0.192	0.767	1.225	2.066	-	0.673	0.190	6.028	3.210	7.505	0.964
n-Triacontane	4.22	4.190	4.368	6.889	4.195	5.111	4.253	0.357	0.219	1.077	1.643	2.606	-	0.827	0.232	8.004	3.636	9.888	1.122
n-Dotriacontane	0.482	2.185	3.287	3.974	1.870	2.923	2.569	0.228	0.345	0.576	1.725	2.005	-	0.109	0.626	5.196	3.118	5.842	0.675
n-Tetratricontane	0.478	1.286	3.303	2.660	1.342	1.965	1.580	0.055	0.346	0.389	0.739	1.040	-	0.333	0.147	4.205	1.454	3.698	0.597
n-Hexatriacontane	0.354	1.114	1.389	2.399	0.933	1.361	1.266	0.030	0.198	0.345	0.538	0.829	-	0.189	0.520	6.129	1.156	2.970	0.586
n-Octatriacontane	0.212	0.578	0.824	1.088	0.555	1.057	0.583	0.018	0.123	0.210	0.339	0.547	-	0.146	0.377	3.558	0.807	2.035	0.428
n-Tetracontane	0.522	13.505	27.755	37.488	25.015	22.588	0.254	0.458	3.359	0.054	11.059	12.521	-	0.114	0.178	139.074	19.790	58.910	18.050
**Depth -8 m**																			
**Compound**	**start**	**17-2**	**17-4**	**17-6**	**17-8**	**18-2**	**18-5**	**19-1**	**21-1**	**23-1**	**23-3**	**24-1**	**24-2**	**24-3**	**24-5**	**27-1**	**28-1**	**28-2**	**29-1**
Naphthalene	0.001	0.015	-	-	-	-	0.060	0.741	0.015	0.934	0.116	0.723	-	-	0.010	0.003	0.005	2.037	0.841
Acenaphthylene	0.016	0.000	-	-	-	-	0.000	0.000	0.000	0.000	0.000	0.000	-	-	0.000	0.000	0.000	0.000	0.000
Acenaphthene	0.000	0.000	-	-	-	-	0.018	0.004	0.001	0.000	0.000	0.000	-	-	0.000	0.000	0.000	0.000	0.036
Fluorene	0.000	0.079	-	-	-	-	0.000	0.000	0.000	0.000	0.000	0.000	-	-	0.000	0.000	0.000	0.000	0.000
Phenanthrene	0.041	0.269	-	-	-	-	0.352	0.198	0.007	0.002	0.000	0.000	-	-	0.181	0.000	0.000	0.299	0.057
Anthracene	0.016	0.000	-	-	-	-	0.130	0.179	0.001	0.000	0.000	0.000	-	-	0.000	0.000	0.000	0.000	0.014
Fluoranthene	0.000	0.000	-	-	-	-	0.000	0.000	0.000	0.000	0.000	0.000	-	-	0.000	0.000	0.000	0.000	0.000
Pyrene	0.000	0.000	-	-	-	-	0.000	0.000	0.000	0.000	0.000	0.000	-	-	0.000	0.000	0.000	0.000	0.000
Chrysene	0.000	0.000	-	-	-	-	0.000	0.000	0.000	0.000	0.000	0.000	-	-	0.000	0.000	0.000	0.000	0.000
Benzo(a)anthracene	0.000	0.000	-	-	-	-	0.000	0.000	0.000	0.000	0.000	0.000	-	-	0.000	0.000	0.000	0.000	0.000
Benzo(b)fluoranthene	0.000	0.000	-	-	-	-	0.000	0.000	0.000	0.000	0.000	0.000	-	-	0.000	0.000	0.000	0.000	0.000

Benzo(k)fluoranthene	0.000	0.000	-	-	-	-	0.000	0.000	0.000	0.000	0.000	0.000	-	-	0.000	0.000	0.000	0.000	0.000
Benzo(a)pyrene	0.000	0.000	-	-	-	-	0.000	0.000	0.000	0.000	0.000	0.000	-	-	0.000	0.000	0.000	0.000	0.000
Indeno(123-cd)pyrene	0.000	0.000	-	-	-	-	0.000	0.000	0.000	0.000	0.000	0.000	-	-	0.000	0.000	0.000	0.000	0.000
Dibenzo(ah)anthracene	0.000	0.000	-	-	-	-	0.000	0.000	0.000	0.000	0.000	0.000	-	-	0.000	0.000	0.000	0.000	0.000
Benzo(ghi)perylen	0.000	0.000	-	-	-	-	0.000	0.000	0.000	0.000	0.000	0.000	-	-	0.000	0.000	0.000	0.000	0.000
2 Methylnaphthalene	0.073	0.133	-	-	-	-	0.066	0.081	0.007	0.239	0.003	0.164	-	-	1.019	0.423	0.116	0.329	0.429
1 Methylnaphthalene	0.075	0.110	-	-	-	-	0.089	0.107	0.007	0.252	0.002	0.164	-	-	0.856	0.377	0.147	0.311	0.439
2,6, Dimethylnaphthalene	0.002	0.062	-	-	-	-	0.000	0.000	0.000	0.000	0.000	0.049	-	-	0.042	0.550	0.057	0.051	0.191
1,6, Dimethylnaphthalene	0.001	0.198	-	-	-	-	0.000	0.000	0.000	0.000	0.000	0.087	-	-	0.720	0.428	0.105	0.123	0.205
1,2, Dimethylnaphthalene	0.000	0.000	-	-	-	-	0.000	0.000	0.000	0.000	0.000	0.000	-	-	0.000	0.000	0.000	0.000	0.000
3 Methylanthracene	0.000	0.000	-	-	-	-	0.000	0.000	0.000	0.000	0.000	0.000	-	-	0.000	0.000	0.000	0.000	0.000
3 Methylphenanthrene	0.000	0.000	-	-	-	-	0.000	0.000	0.000	0.000	0.000	0.000	-	-	0.000	0.000	0.000	0.000	0.000
2 Methylphenanthrene	0.000	0.000	-	-	-	-	0.000	0.000	0.000	0.000	0.000	0.000	-	-	0.000	0.000	0.000	0.000	0.000
9 Methylphenanthrene	0.000	0.000	-	-	-	-	0.000	0.000	0.000	0.000	0.000	0.000	-	-	0.000	0.000	0.000	0.000	0.000
1 Methylphenanthrene	0.000	0.000	-	-	-	-	0.000	0.000	0.000	0.000	0.000	0.000	-	-	0.000	0.000	0.000	0.000	0.000
1,7 Dimethylphenanthrene	0.000	0.000	-	-	-	-	0.000	0.000	0.000	0.197	0.000	0.000	-	-	0.002	0.000	0.000	0.000	0.000
1+3 Methylfluoranthene	0.000	0.000	-	-	-	-	0.000	0.000	0.000	0.000	0.000	0.000	-	-	0.000	0.000	0.000	0.000	0.000
Retene	0.000	0.000	-	-	-	-	0.000	0.000	0.000	0.000	0.000	0.000	-	-	0.000	0.000	0.000	0.000	0.000
3 Methylpyrene	0.000	0.000	-	-	-	-	0.000	0.000	0.000	0.000	0.000	0.000	-	-	0.000	0.000	0.000	0.000	0.000
1 Methylpyrene	0.000	0.000	-	-	-	-	0.000	0.000	0.000	0.000	0.000	0.000	-	-	0.000	0.000	0.000	0.000	0.000
3 Methylchrysene	0.000	0.000	-	-	-	-	0.000	0.000	0.000	0.000	0.000	0.000	-	-	0.000	0.000	0.000	0.000	0.000
6 Methylchrysene	0.000	0.000	-	-	-	-	0.000	0.000	0.000	0.000	0.000	0.000	-	-	0.000	0.000	0.000	0.000	0.000
n-Decane	0.098	0.235	-	-	-	-	0.524	1.682	0.002	3.202	0.000	0.008	-	-	0.054	0.101	0.000	0.000	0.362
n-Dodecane	0.587	0.210	-	-	-	-	3.624	3.246	0.228	3.435	0.000	0.423	-	-	0.098	0.435	0.530	0.879	3.095
n-Tetradecane	0.452	0.817	-	-	-	-	7.646	10.203	1.524	2.576	0.065	3.834	-	-	0.625	0.254	2.707	3.253	9.482
n-Hexadecane	0.048	0.075	-	-	-	-	0.188	0.224	0.238	0.384	0.017	0.331	-	-	0.058	0.000	0.388	0.481	1.018
n-Heptadecane	0.015	0.040	-	-	-	-	3.433	2.565	0.001	0.446	0.000	0.000	-	-	0.000	0.000	0.000	0.000	0.124
Pristane	0.012	0.007	-	-	-	-	3.399	2.528	0.002	0.458	0.000	0.000	-	-	0.000	0.000	0.000	0.000	0.768
n-Octadecane	0.034	0.046	-	-	-	-	1.093	0.172	0.001	0.425	0.000	0.000	-	-	0.020	0.000	0.000	0.000	0.131
Phytane	0.028	0.009	-	-	-	-	1.098	1.021	0.002	1.073	0.000	0.000	-	-	0.001	0.000	0.000	0.000	0.381
n-Eicosane	0.042	0.071	-	-	-	-	0.233	0.395	0.001	0.003	0.000	0.000	-	-	0.016	0.000	0.000	0.005	0.315
n-Docosane	0.115	0.322	-	-	-	-	0.294	0.764	0.155	0.575	0.018	0.057	-	-	0.072	0.024	0.032	0.024	0.880
n-Tetracosane	0.429	2.148	-	-	-	-	1.467	3.054	0.528	3.743	0.096	0.783	-	-	1.134	0.148	0.854	0.760	1.421
n-Hexacosane	0.594	2.634	-	-	-	-	1.378	2.381	1.259	9.499	0.135	1.621	-	-	3.629	0.357	1.661	1.501	1.088
n-Octacosane	1.048	3.213	-	-	-	-	2.269	3.109	3.468	14.282	0.089	2.868	-	-	8.303	0.632	2.896	3.014	1.873
n-Triacontane	1.396	3.116	-	-	-	-	2.718	3.338	5.500	16.450	0.086	3.773	-	-	12.702	0.780	3.922	4.319	2.417
n-Dotriacontane	0.670	1.645	-	-	-	-	1.405	1.603	3.152	9.355	0.119	2.329	-	-	4.663	0.494	2.322	2.928	1.357
n-Tetratricontane	0.398	0.824	-	-	-	-	1.033	1.126	2.186	7.996	0.097	1.537	-	-	7.894	0.312	1.630	1.982	1.022
n-Hexatriacontane	0.337	0.632	-	-	-	-	1.553	1.294	1.667	7.727	0.057	1.026	-	-	0.974	0.051	1.328	1.750	1.932
n-Octatriacontane	0.157	0.437	-	-	-	-	0.496	0.490	1.203	2.360	0.000	0.609	-	-	4.365	0.181	0.919	1.191	0.453
n-Tetracontane	1.712	10.108	-	-	-	-	8.349	32.171	39.198	64.332	0.000	11.227	-	-	5.787	0.109	23.391	33.459	8.098

Supplementary Table 4. Hydrocarbon analysis in seawater samples at the ES-2009 spill at 1.5m depth.

	Inside slick									Slick Edge			Outside slick					
Time (h)	0.000	3.090	16.800	16.800	19.100	19.100	19.100	22.100	24.700	0.000	16.800	19.100	0.000	3.090	16.840	19.070	22.070	24.730
Conc µg/l	M 2 I 1.5m	M 3 I 1.5m	M 4 I 1.5m	M 4 I,5m / 2	M 5 I 1.5m/1 S1	M 5 I,5m /1 S2	M 5 I,5m / 2	M 6 I 1,5m	M 7 I 1,5m	M2 E 1.5m	M 4 E 1.5m	M 5 E 1.5m	M 2 O 1.5m	M 3 O 1.5m	M 4 O 1.5m	M 5 O 1.5m	M 6 O 1.5m	M 7 O 1.5m
Naphthalene	0.042	0.189	0.666	0.721	0.670	0.238	0.748	0.343	0.248	0.034	0.250	0.503	0.062	0.185	0.229	0.413	0.367	0.178
2-Methylnaphthalene	0.021	0.084	0.138	0.605	0.597	0.072	0.556	0.099	0.114	0.011	0.102	0.451	0.000	0.066	0.114	0.161	0.132	0.070
1-Methylnaphthalene	0.006	0.045	0.092	0.424	0.402	0.049	0.320	0.067	0.080	0.006	0.068	0.275	0.000	0.037	0.061	0.093	0.085	0.048
2.6 Dimethylnaphthalene	0.004	0.013	0.024	0.011	0.000	0.012	0.078	0.017	0.024	0.001	0.019	0.003	0.000	0.008	0.013	0.030	0.027	0.013
1.6 Dimethylnaphthalene	0.002	0.009	0.019	0.008	0.000	0.010	0.073	0.009	0.016	0.001	0.015	0.002	0.000	0.006	0.012	0.022	0.019	0.009
1.2 Dimethylnaphthalene	0.000	0.000	0.000	0.000	0.000	0.000	0.000	0.000	0.000	0.000	0.000	0.000	0.000	0.000	0.000	0.000	0.000	0.000
Acenaphthylene	0.000	0.004	0.003	0.008	0.000	0.004	0.009	0.001	0.002	0.003	0.001	0.000	0.000	0.004	0.002	0.004	0.008	0.004
Acenaphthene	0.001	0.019	0.005	0.007	0.000	0.012	0.013	0.003	0.000	0.001	0.006	0.001	0.000	0.008	0.013	0.011	0.000	0.005
Fluorene	0.017	0.228	0.290	0.179	0.017	0.312	0.004	0.320	0.318	0.025	0.276	0.026	0.000	0.195	0.311	0.386	0.406	0.228
Phenanthrene	0.005	0.067	0.055	0.134	0.195	0.335	0.088	0.693	0.048	0.006	0.052	0.055	0.000	0.040	0.086	0.064	0.082	0.046
2-Methylphenanthrene	0.001	0.005	0.004	0.000	0.000	0.000	0.000	0.000	0.000	0.000	0.000	0.000	0.000	0.000	0.000	0.000	0.000	0.000
1-Methylphenanthrene	0.001	0.003	0.005	0.000	0.000	0.000	0.000	0.000	0.000	0.000	0.000	0.000	0.000	0.000	0.000	0.000	0.000	0.000
Anthracene	0.002	0.035	0.021	0.028	0.000	0.103	0.000	0.000	0.005	0.002	0.056	0.002	0.000	0.037	0.071	0.038	0.000	0.007
2- Methylanthracene	0.000	0.000	0.000	0.000	0.000	0.000	0.000	0.000	0.000	0.000	0.000	0.000	0.000	0.000	0.000	0.000	0.000	0.000
Fluoanthene	0.002	0.000	0.005	0.000	0.000	0.000	0.000	0.000	0.005	0.002	0.000	0.001	0.000	0.000	0.000	0.000	0.000	0.005
Pyrene	0.002	0.000	0.008	0.000	0.000	0.009	0.000	0.001	0.006	0.003	0.000	0.001	0.000	0.000	0.000	0.001	0.000	0.010
Chrysene	0.000	0.000	0.001	0.000	0.000	0.000	0.000	0.000	0.000	0.000	0.000	0.000	0.000	0.000	0.000	0.000	0.000	0.000
6-Methylchrysene	0.000	0.000	0.000	0.000	0.000	0.000	0.000	0.000	0.000	0.000	0.000	0.000	0.000	0.000	0.000	0.000	0.000	0.000
Benzo(a)anthracene	0.000	0.000	0.001	0.000	0.000	0.000	0.000	0.000	0.000	0.000	0.000	0.000	0.000	0.000	0.000	0.000	0.000	0.000
Benzo(b)fluoranthen	0.000	0.000	0.000	0.000	0.000	0.000	0.000	0.000	0.000	0.000	0.000	0.000	0.000	0.000	0.000	0.000	0.000	0.000
Benzo(k)fluoranthen	0.000	0.000	0.000	0.000	0.000	0.000	0.000	0.000	0.000	0.000	0.000	0.000	0.000	0.000	0.000	0.000	0.000	0.000
Benzo(a)pyrene	0.000	0.000	0.000	0.000	0.000	0.000	0.000	0.000	0.000	0.000	0.000	0.000	0.000	0.000	0.000	0.000	0.000	0.000
Indeno(1,2,3,cd)pyrene	0.000	0.000	0.000	0.000	0.000	0.000	0.000	0.000	0.000	0.000	0.000	0.000	0.000	0.000	0.000	0.000	0.000	0.000
Dibenzo(a,h)anthracene	0.000	0.000	0.000	0.000	0.000	0.000	0.000	0.000	0.000	0.000	0.000	0.000	0.000	0.000	0.000	0.000	0.000	0.000
Benzo(g,h,i)prylene	0.000	0.000	0.000	0.000	0.000	0.000	0.000	0.000	0.000	0.000	0.000	0.000	0.000	0.000	0.000	0.000	0.000	0.000
n - Hexane	0.000	0.000	0.000	0.000	0.000	0.000	0.000	0.000	0.000	0.000	0.000	0.000	0.000	0.000	0.000	0.000	0.000	0.000
n - Heptane	0.000	0.000	0.000	0.000	0.000	0.000	0.000	0.000	0.000	0.000	0.000	0.000	0.000	0.000	0.000	0.000	0.000	0.000
n-Octane	2.300	1.761	10.062	0.000	4.706	8.781	2.750	14.992	14.626	0.000	0.000	5.496	0.295	1.039	4.701	2.240	6.727	19.039
n-Nonane	0.000	0.000	0.000	0.000	0.000	0.297	0.122	1.887	0.000	0.000	0.000	0.000	0.000	0.000	0.000	0.070	4.668	0.476
n-Decane	5.659	5.748	4.921	2.349	4.205	4.665	4.828	6.216	6.141	4.439	5.627	4.609	3.287	0.312	2.959	5.288	6.485	7.052
n-Undecane	1.349	1.256	2.065	1.237	0.059	0.967	1.160	0.860	0.821	1.285	0.728	1.660	1.283	0.761	1.408	2.354	0.617	0.899
n-Dodecane	0.476	0.378	1.434	1.165	0.572	1.391	1.493	2.164	0.919	0.528	0.655	0.792	1.117	0.561	0.971	1.504	1.377	2.789
n-Tetradecane	0.062	1.204	0.363	2.073	3.450	3.731	2.558	4.682	2.579	0.000	0.859	1.298	0.114	1.236	3.748	7.423	5.362	3.428
n-Hexadecane	0.000	0.000	0.085	1.011	0.000	0.000	2.260	1.634	1.318	0.026	0.000	0.424	0.000	0.000	0.000	0.702	2.519	1.067

n-Heptadecane	0.000	0.000	0.076	0.901	0.000	0.000	1.347	1.209	0.000	0.000	0.000	0.280	0.000	0.000	0.000	0.000	0.000	0.000
Pristane	0.000	0.000	0.022	0.000	0.000	0.000	0.125	0.000	0.000	0.000	0.000	0.000	0.000	0.000	0.000	0.000	0.000	0.000
n-Octadecane	0.000	0.000	0.046	0.483	0.000	0.000	0.000	0.000	0.000	0.000	0.000	0.000	0.000	0.000	0.000	0.000	0.000	0.000
Phytane	0.000	0.000	0.023	0.000	0.000	0.000	0.000	0.000	0.000	0.000	0.000	0.000	0.000	0.000	0.000	0.000	0.000	0.000
n-Eicosane	0.000	0.000	0.025	0.795	0.000	0.000	0.511	0.829	0.687	0.000	0.000	0.000	0.000	0.000	0.000	0.000	0.000	0.000
n-Docosane	0.058	0.000	0.151	2.669	2.983	1.661	1.052	1.553	1.820	0.000	0.000	0.235	0.000	0.076	0.000	0.746	2.600	1.602
n-Tetracosane	0.064	0.356	0.209	4.452	2.374	3.171	3.480	1.732	2.148	0.017	0.427	0.390	0.000	0.138	0.226	1.618	2.386	1.564
n-Hexacosane	0.000	0.043	0.097	1.333	1.287	1.667	1.431	0.480	0.879	0.000	0.188	0.101	0.000	0.149	0.000	1.105	1.893	1.055
n-Octacosane	0.072	0.365	0.106	0.864	0.758	0.900	0.834	0.000	1.227	0.025	0.186	0.176	0.000	0.314	0.376	0.702	0.000	0.000
n-Triacontane	0.087	0.341	0.211	1.064	0.000	0.694	2.818	0.403	1.750	0.000	0.426	0.233	0.000	0.580	0.000	0.000	0.000	0.000
n-Dotriacontane	0.083	0.000	0.137	0.000	0.000	0.000	1.157	0.000	1.192	0.000	0.000	0.000	0.000	0.513	0.000	0.000	0.000	0.000
n-Tetratricontane	0.000	0.000	0.106	0.000	0.000	0.000	0.000	0.000	0.000	0.000	0.000	0.000	0.000	0.000	0.000	0.000	0.000	0.000
n-Hexatriacontane	0.000	0.000	0.075	0.000	0.000	0.000	0.000	0.000	0.000	0.000	0.000	0.000	0.000	0.000	0.000	0.000	0.000	0.000
n-Octatriacontane	0.000	0.000	0.000	0.000	0.000	0.000	0.000	0.000	0.000	0.000	0.000	0.000	0.000	0.000	0.000	0.000	0.000	0.000
n-Tetracontane	0.000	0.000	0.000	0.000	0.000	0.000	0.000	0.000	0.000	0.000	0.000	0.000	0.000	0.000	0.000	0.000	0.000	0.000
Benzene	0.000	0.000	0.000	0.000	0.000	0.000	0.000	0.000	0.000	0.000	0.000	0.000	0.000	0.000	0.168	0.000	0.000	0.000
Toluene	4.814	0.871	99.766	10.190	31.785	1.408	1.278	33.690	42.743	1.443	2.254	56.187	0.000	2.205	1.172	1.242	1.800	0.646
p + m Xylene	0.000	0.000	0.000	0.000	0.000	0.000	0.000	0.000	0.000	0.000	0.000	0.000	0.000	0.000	0.000	0.000	0.000	0.000
Ethylbenzene	0.000	0.000	0.000	0.000	0.000	0.000	0.000	0.000	0.000	0.000	0.000	0.000	0.000	0.000	0.000	0.000	0.000	0.000
o - Xylene	0.000	0.000	0.000	0.000	0.000	0.000	0.000	0.000	0.000	0.000	0.000	0.000	0.000	0.000	0.000	0.000	0.000	0.000

Supplementary Table 5. Hydrocarbon analysis in seawater samples at the ES-2009 spill at 8 m depth.

	Inside slick							Slick edge		Outside slick					
Time (h)	0.000	3.090	16.800	19.100	19.100	22.100	24.700	3.090	16.800	0.000	3.090	16.840	19.070	22.070	24.730
Concentration (µg/)l	M 2 I 8m	M 3 I 8m	M 4 I 8m	M 5 I/2 8m	M 5 I/1 8m	M 6 I 8m	M 7 I 8m	M 3 E 8m	M 4 E 8m	M 2 O 8m	M 3 O 8m	M 4 O 8m	M 5 O 8m	M6 O 8m	M 7 O 8m
Naphthalene	0.055	0.242	0.244	0.337	0.340	0.270	0.218	0.188	0.201	0.058	0.209	0.232	0.504	0.242	0.171
2-Methylnaphthalene	0.023	0.099	0.126	0.121	0.119	0.090	0.071	0.081	0.100	0.000	0.082	0.115	0.174	0.081	0.081
1-Methylnaphthalene	0.012	0.062	0.077	0.076	0.078	0.066	0.047	0.042	0.057	0.000	0.040	0.076	0.101	0.051	0.054
2.6 Dimethylnaphthalene	0.006	0.068	0.035	0.025	0.022	0.019	0.014	0.010	0.021	0.000	0.009	0.023	0.032	0.014	0.017
1.6 Dimethylnaphthalene	0.006	0.023	0.028	0.009	0.017	0.015	0.009	0.007	0.012	0.000	0.008	0.014	0.024	0.008	0.016
1.2 Dimethylnaphthalene	0.000	0.000	0.000	0.000	0.014	0.006	0.000	0.000	0.000	0.000	0.000	0.000	0.000	0.000	0.006
Acenaphthylene	0.006	0.004	0.005	0.003	0.003	0.003	0.002	0.003	0.005	0.000	0.002	0.005	0.005	0.004	0.004
Acenaphthene	0.002	0.022	0.015	0.018	0.008	0.004	0.007	0.006	0.011	0.000	0.012	0.000	0.012	0.005	0.008
Fluorene	0.039	0.226	0.328	0.456	0.361	0.278	0.182	0.224	0.258	0.000	0.226	0.291	0.401	0.244	0.198
Phenanthrene	0.014	0.129	0.106	0.131	0.093	0.056	0.033	0.048	0.053	0.000	0.029	0.053	0.068	0.047	0.042
2-Methylphenanthrene	0.003	0.000	0.000	0.000	0.000	0.000	0.000	0.000	0.000	0.000	0.000	0.000	0.004	0.000	0.000
1-Methylphenanthrene	0.002	0.000	0.000	0.000	0.000	0.000	0.000	0.000	0.000	0.000	0.000	0.000	0.002	0.000	0.000
Anthracene	0.004	0.048	0.035	0.070	0.014	0.012	0.004	0.037	0.048	0.000	0.015	0.058	0.028	0.009	0.007
2- Methylanthracene	0.000	0.000	0.000	0.000	0.000	0.000	0.000	0.000	0.000	0.000	0.000	0.000	0.000	0.000	0.000
Fluoanthen	0.005	0.000	0.000	0.023	0.000	0.003	0.004	0.000	0.000	0.000	0.000	0.000	0.006	0.004	0.007
Pyrene	0.007	0.000	0.000	0.012	0.000	0.010	0.005	0.000	0.000	0.000	0.000	0.000	0.012	0.007	0.002
Chrysene	0.000	0.000	0.000	0.000	0.000	0.000	0.001	0.000	0.000	0.000	0.000	0.000	0.012	0.000	0.000
6-Methylchrysene	0.000	0.000	0.000	0.000	0.000	0.000	0.000	0.000	0.000	0.000	0.000	0.000	0.000	0.000	0.000
Benzo(a)anthracene	0.000	0.000	0.000	0.000	0.000	0.000	0.001	0.000	0.000	0.000	0.000	0.000	0.000	0.000	0.000
Benzo(b)fluoranthen	0.000	0.000	0.000	0.000	0.000	0.000	0.000	0.000	0.000	0.000	0.000	0.000	0.000	0.000	0.000
Benzo(k)fluoranthen	0.000	0.000	0.000	0.000	0.000	0.000	0.000	0.000	0.000	0.000	0.000	0.000	0.000	0.000	0.000
Benzo(a)pyrene	0.000	0.000	0.000	0.000	0.000	0.000	0.000	0.000	0.000	0.000	0.000	0.000	0.000	0.000	0.000
Indeno(1,2,3,cd)pyrene	0.000	0.000	0.000	0.000	0.000	0.000	0.000	0.000	0.000	0.000	0.000	0.000	0.000	0.000	0.000
Dibenzo(a,h)anthracene	0.000	0.000	0.000	0.000	0.000	0.000	0.000	0.000	0.000	0.000	0.000	0.000	0.000	0.000	0.000
Benzo(g,h,i)prylene	0.000	0.000	0.000	0.000	0.000	0.000	0.000	0.000	0.000	0.000	0.000	0.000	0.000	0.000	0.000
n - Hexane	0.000	0.000	0.000	0.000	0.000	0.000	0.000	0.000	0.000	0.000	0.000	0.000	0.000	0.000	0.000
n - Heptane	0.000	0.000	0.000	0.000	0.000	0.000	0.000	0.000	0.000	0.000	0.000	0.000	0.000	0.000	0.000
n-Octane	1.542	2.626	9.330	13.994	13.330	14.277	9.332	3.486	5.338	0.000	0.000	0.000	0.000	0.000	0.000
n-Nonane	0.000	0.170	0.000	0.000	0.157	0.106	0.000	0.000	0.000	0.558	0.080	0.000	0.000	0.708	0.970
n-Decane	6.018	4.587	4.766	5.707	5.338	8.411	4.535	2.053	4.790	4.772	5.786	4.454	3.953	5.423	6.336
n-Undecane	2.629	1.580	2.054	0.610	2.654	1.538	1.035	0.576	2.028	1.106	3.002	1.833	0.384	0.821	1.691
n-Dodecane	1.291	0.562	1.531	0.869	1.839	1.350	0.264	0.496	0.548	2.170	0.882	1.422	0.500	0.376	1.992
n-Tetradecane	0.164	1.859	0.905	3.559	3.711	2.821	2.662	1.286	1.695	0.000	0.895	2.338	4.334	4.950	2.398
n-Hexadecane	0.000	0.000	0.205	1.837	0.122	1.173	1.564	0.000	0.000	0.000	0.109	0.000	2.543	0.922	1.446

n-Heptadecane	0.000	0.000	0.158	0.000	0.000	1.886	0.752	0.000	0.000	0.000	0.000	0.000	0.000	0.000	1.097
Pristane	0.000	0.000	0.000	0.000	0.000	0.000	0.098	0.000	0.000	0.000	0.000	0.000	0.000	0.000	0.000
n-Octadecane	0.000	0.000	0.000	0.000	0.000	0.238	0.000	0.000	0.000	0.000	0.000	0.000	0.000	0.000	0.000
Phytane	0.000	0.000	0.000	0.000	0.000	0.000	0.000	0.000	0.000	0.000	0.000	0.000	0.000	0.000	0.000
n-Eicosane	0.000	0.000	0.000	0.000	0.000	0.781	0.636	0.000	0.000	0.000	0.000	0.000	0.000	0.000	1.020
n-Docosane	0.022	0.000	0.421	1.236	2.045	1.619	1.740	0.129	0.000	0.000	0.040	0.000	0.601	2.316	1.481
n-Tetracosane	0.047	0.000	0.543	0.563	2.340	1.976	2.353	0.231	0.000	0.000	0.127	0.000	2.634	3.359	1.300
n-Hexacosane	0.000	0.000	0.465	1.792	0.306	0.514	1.264	0.066	0.000	0.000	0.081	0.000	0.355	0.000	0.322
n-Octacosane	0.000	0.000	0.601	2.500	0.859	0.627	1.319	0.000	0.000	0.000	0.065	0.000	0.394	0.283	0.570
n-Triacontane	0.000	0.000	0.462	1.799	0.000	0.000	2.491	0.220	0.000	0.000	0.000	0.000	0.572	0.000	0.794
n-Dotriacontane	0.000	0.000	0.000	0.000	0.000	0.000	0.501	0.000	0.000	0.000	0.000	0.000	0.589	0.000	0.555
n-Tetratricontane	0.000	0.000	0.000	0.000	0.000	0.000	0.000	0.000	0.000	0.000	0.000	0.000	0.000	0.000	0.000
n-Hexatriacontane	0.000	0.000	0.000	0.000	0.000	0.000	0.000	0.000	0.000	0.000	0.000	0.000	0.000	0.000	0.000
n-Octatriacontane	0.000	0.000	0.000	0.000	0.000	0.000	0.000	0.000	0.000	0.000	0.000	0.000	0.000	0.000	0.000
n-Tetracontane	0.000	0.000	0.000	0.000	0.000	0.000	0.000	0.000	0.000	0.000	0.000	0.000	0.000	0.000	0.000
Benzene	0.000	0.032	0.000	0.000	0.000	0.000	0.000	0.000	0.000	0.000	0.000	0.000	0.146	0.000	0.000
Toluene	1.007	5.198	3.643	33.353	1.274	2.364	2.016	0.862	1.774	0.860	0.600	0.983	1.756	2.948	0.750
p + m Xylene	0.000	0.000	0.000	0.000	0.000	0.000	0.000	0.000	0.000	0.000	0.000	0.000	0.000	0.000	0.000
Ethylbenzene	0.000	0.000	0.000	0.000	0.000	0.000	0.000	0.000	0.000	0.000	0.000	0.000	0.000	0.000	0.000
o - Xylene	0.000	0.000	0.000	0.000	0.000	0.000	0.000	0.000	0.000	0.000	0.000	0.000	0.000	0.000	0.000

Supplementary Table 6. Hydrocarbon concentrations in seawater samples from oiled mesocosm C-III

Conc µg/l	Time(h)	**0**	**2**	**6**	**11**	**29**	**52**	**76**	**100**	**124**	**148**	**172**
TOP		III wo 0	III wo 1	III wo 2	III wo3	III Wo 4	III Wo 5	III Wo 6				
Naphthalene		0.028	0.911	1.080	2.534	11.641	21.494	92.337				
Acenaphthylene		0.000	0.000	0.000	0.000	0.000	0.000	0.000				
Acenaphthene		0.000	0.000	0.000	0.001	0.001	0.007	0.000				
Fluorene		0.001	0.000	0.000	0.001	0.001	0.065	0.000				
Phenanthrene		0.006	0.001	0.000	0.003	0.260	0.154	0.003				
Anthracene		0.000	0.000	0.009	0.003	0.003	0.092	0.000				
Fluoranthene		0.000	0.000	0.000	0.000	0.000	0.118	0.000				
Pyrene		0.000	0.000	0.000	0.000	0.000	0.000	0.000				
Chrysene		0.000	0.000	0.000	0.000	0.000	0.000	0.000				
Benzo(a)anthracene		0.000	0.000	0.000	0.000	0.000	0.000	0.000				
Benzo(b)fluoranthene		0.000	0.000	0.000	0.000	0.000	0.000	0.000				
Benzo(k)fluoranthene		0.000	0.000	0.000	0.000	0.000	0.000	0.000				
Benzo(a)pyrene		0.000	0.000	0.000	0.000	0.000	0.000	0.000				
Indeno(123-cd)pyrene		0.000	0.000	0.000	0.000	0.000	0.000	0.000				
Dibenzo(ah)anthracene		0.004	0.000	0.000	0.000	0.000	0.000	0.000				
Benzo(ghi)perylen		0.004	0.000	0.000	0.000	0.000	0.000	0.000				
2 Methylnaphthalene		0.008	0.061	0.050	0.063	0.347	0.929	4.800				
1 Methylnaphthalene		0.004	0.048	0.039	0.050	0.409	0.898	4.026				
2,6, Dimethylnaph		0.004	0.000	0.000	0.001	0.001	0.022	0.140				
1,6, Dimethylnaphthalene		0.102	0.000	0.001	0.001	0.002	0.035	0.165				
1,2, Dimethylnaphthalene		0.000	0.000	0.000	0.000	0.000	0.011	0.101				
3 Methylanthracene		0.000	0.000	0.000	0.000	0.000	0.003	0.000				
3 Methylphenanthrene		0.000	0.000	0.000	0.000	0.000	0.000	0.000				
2 Methylphenanthrene		0.000	0.000	0.000	0.000	0.000	0.000	0.000				
9 Methylphenanthrene		0.000	0.000	0.000	0.000	0.000	0.000	0.000				
1 Methylphenanthrene		0.000	0.000	0.000	0.000	0.000	0.000	0.000				
1,7 Dimethylphenanthrene		0.000	0.000	0.000	0.000	0.000	0.007	0.000				
1+3 Methylfluoranthene		0.000	0.000	0.000	0.000	0.000	0.000	0.000				
Retene		0.000	0.000	0.000	0.000	0.000	0.000	0.000				
3 Methylpyrene		0.000	0.000	0.000	0.000	0.000	0.001	0.000				
1 Methylpyrene		0.000	0.000	0.000	0.000	0.000	0.000	0.000				
3 Methylchrysene		0.000	0.000	0.000	0.000	0.000	0.002	0.000				
6 Methylchrysene		0.000	0.000	0.000	0.000	0.000	0.000	0.000				
n-Decane		0.000	0.334	0.271	0.401	0.957	0.172	0.143				
n-Dodecane		0.004	6.009	6.508	1.830	1.022	1.331	55.163				
n-Tetradecane		0.064	1.267	1.495	0.599	0.649	0.290	11.751				
n-Hexadecane		0.014	0.002	0.084	0.003	0.235	0.097	0.001				
n-Heptadecane		0.011	0.000	0.000	0.005	0.131	0.099	0.000				
Pristane		0.000	0.000	0.000	0.000	0.102	0.145	0.000				
n-Octadecane		0.011	0.000	0.002	0.000	2.305	0.262	0.000				
Phytane		0.000	0.000	0.000	0.000	0.918	0.273	0.000				
n-Eicosane		0.002	0.000	0.001	0.005	0.278	0.106	0.004				
n-Docosane		0.008	0.069	0.250	0.020	0.404	0.181	1.665				
n-Tetracosane		0.039	0.387	0.986	0.179	1.631	0.881	6.958				
n-Hexacosane		0.028	0.636	1.046	0.226	3.367	2.762	6.679				
n-Octacosane		0.054	0.478	0.847	0.243	2.728	14.555	5.497				
n-Triacontane		0.076	0.417	0.690	0.158	9.451	27.210	4.136				
n-Dotriacontane		0.053	0.445	0.558	0.121	4.919	13.377	3.320				
n-Tetratricontane		0.044	0.420	0.387	0.093	3.500	9.863	2.315				
n-Hexatriacontane		0.033	0.235	0.192	0.042	2.974	8.078	1.151				
n-Octatriacontane		0.026	0.142	0.115	0.026	1.936	3.957	0.497				
n-Tetracontane		0.000	0.088	0.000	0.000	1.059	1.706	0.000				
BOTTOM		III wu 0	III wu 1	III wu 2	III wu 3	III Wu 4	III Wu 5	III Wu 6	III Wu 7	III Wu 8	III Wu 9	III Wu 10
Naphthalene		0.031	0.031	1.143	3.238	16.423	35.678	-	71.738	59.927	107.131	107.414

Acenaphthylene		0.000	0.000	0.000	0.000	0.000	0.000	-	0.000	0.000	0.000	0.000
Acenaphthene		0.000	0.000	0.000	0.000	0.001	0.000	-	0.000	0.000	0.000	0.154
Fluorene		0.001	0.007	0.000	0.000	0.007	0.002	-	0.011	0.175	0.032	0.154
Phenanthrene		0.007	0.003	0.002	0.007	0.353	0.353	-	0.454	0.520	0.540	0.211
Anthracene		0.000	0.003	0.003	0.000	0.003	0.246	-	0.376	0.460	0.544	0.008
Fluoranthene		0.000	0.000	0.000	0.000	0.000	0.000	-	0.000	0.008	0.000	0.000
Pyrene		0.000	0.000	0.000	0.000	0.000	0.000	-	0.000	0.000	0.001	0.000
Chrysene		0.000	0.000	0.000	0.000	0.000	0.000	-	0.000	0.000	0.000	0.000
Benzo(a)anthracene		0.000	0.000	0.000	0.000	0.000	0.000	-	0.000	0.000	0.000	0.000
Benzo(b)fluoranthene		0.000	0.000	0.000	0.000	0.000	0.000	-	0.000	0.000	0.000	0.000
Benzo(k)fluoranthene		0.000	0.000	0.000	0.000	0.000	0.000	-	0.000	0.000	0.000	0.000
Benzo(a)pyrene		0.000	0.000	0.000	0.000	0.000	0.000	-	0.000	0.000	0.000	0.000
Indeno(123-cd)pyrene		0.000	0.000	0.000	0.000	0.000	0.000	-	0.000	0.000	0.000	0.000
Dibenzo(ah)anthracene		0.001	0.000	0.000	0.000	0.000	0.000	-	0.000	0.000	0.000	0.000
Benzo(ghi)perylen		0.001	0.000	0.000	0.000	0.000	0.000	-	0.000	0.000	0.000	0.000
2 Methylnaphthalene		0.009	0.050	0.054	0.048	1.413	2.828	-	7.776	6.758	6.778	5.167
1 Methylnaphthalene		0.005	0.037	0.042	0.030	1.363	2.883	-	7.515	6.853	26.100	6.005
2,6, Dimethylnaph		0.004	0.000	0.000	0.000	0.068	0.053	-	0.606	0.564	1.169	0.441
1,6, Dimethylnaphthalene		0.093	0.000	0.000	0.000	0.120	0.093	-	0.830	0.818	1.630	0.745
1,2, Dimethylnaphthalene		0.000	0.000	0.022	0.000	0.033	0.025	-	0.265	0.278	0.654	0.238
3 Methylanthracene		0.015	0.000	0.000	0.000	0.000	0.070	-	0.039	0.040	0.264	0.022
3 Methylphenanthrene		0.000	0.000	0.000	0.000	0.000	0.000	-	0.000	0.000	0.003	0.036
2 Methylphenanthrene		0.000	0.000	0.000	0.000	0.000	0.000	-	0.000	0.000	0.004	0.019
9 Methylphenanthrene		0.000	0.000	0.000	0.000	0.000	0.000	-	0.000	0.001	0.010	0.022
1 Methylphenanthrene		0.000	0.000	0.000	0.000	0.000	0.000	-	0.000	0.001	0.005	0.053
1,7 Dimethylphenanthrene		0.000	0.000	0.000	0.000	0.000	0.000	-	0.139	0.193	0.716	0.000
1+3 Methylfluoranthene		0.000	0.000	0.000	0.000	0.000	0.000	-	0.000	0.000	0.000	0.000
Retene		0.000	0.000	0.000	0.000	0.000	0.000	-	0.000	0.000	0.000	0.000
3 Methylpyrene		0.000	0.000	0.000	0.000	0.000	0.000	-	0.013	0.017	0.051	0.000
1 Methylpyrene		0.000	0.000	0.000	0.000	0.000	0.000	-	0.000	0.137	0.000	0.000
3 Methylchrysene		0.000	0.000	0.000	0.000	0.000	0.000	-	0.000	0.000	0.000	0.000
6 Methylchrysene		0.000	0.000	0.000	0.000	0.000	0.000	-	0.000	0.000	0.000	0.000
n-Decane		0.000	0.428	5.541	4.120	0.630	2.204	-	1.278	0.752	0.279	0.004
n-Dodecane		0.010	5.085	8.341	3.138	2.519	3.302	-	1.407	1.309	0.527	0.434
n-Tetradecane		0.673	1.059	1.756	0.647	1.159	1.518	-	1.453	1.484	0.667	1.122
n-Hexadecane		0.015	0.095	0.002	0.022	0.299	0.737	-	9.358	5.327	2.459	0.004
n-Heptadecane		0.002	0.002	0.005	0.010	0.273	0.277	-	1.122	1.502	0.758	0.002
Pristane		0.000	0.002	0.003	0.002	0.194	0.203	-	1.026	0.480	0.165	0.000
n-Octadecane		0.002	0.005	0.003	0.008	0.341	0.243	-	0.666	0.521	0.252	0.013
Phytane		0.000	0.001	0.000	0.003	0.072	0.008	-	0.005	0.007	0.223	0.000
n-Eicosane		0.012	0.003	0.004	0.005	0.593	0.459	-	1.034	0.002	0.002	0.001
n-Docosane		0.031	0.031	0.006	0.027	2.401	0.903	-	3.526	0.391	1.327	0.006
n-Tetracosane		0.080	0.237	0.420	0.227	7.410	8.940	-	7.419	2.228	3.271	0.073
n-Hexacosane		0.084	0.457	0.524	0.395	6.505	4.478	-	5.960	1.584	2.266	0.101
n-Octacosane		0.115	0.404	0.475	0.313	7.252	7.754	-	6.203	1.982	1.847	0.126
n-Triacontane		0.115	0.340	0.394	0.258	6.923	7.450	-	5.586	2.660	1.329	0.134
n-Dotriacontane		0.109	0.251	0.283	0.190	3.411	0.877	-	3.056	2.518	0.710	0.053
n-Tetratricontane		0.096	0.167	0.186	0.120	1.999	2.820	-	2.177	2.918	0.576	0.042
n-Hexatriacontane		0.067	0.088	0.117	0.070	1.750	4.821	-	1.300	1.643	0.375	0.038
n-Octatriacontane		0.038	0.000	0.005	0.043	1.128	2.421	-	0.821	1.139	0.251	0.014
n-Tetracontane		0.000	0.000	0.000	0.004	0.427	0.153	-	0.351	0.636	0.151	0.151

Supplementary References

1. Wang, Z. *et al.* Characteristics of Spilled Oils, Fuels, and Petroleum Products:1. Composition and Properties of Selected Oils. (Emergencies Science and Technology Division, Environmental Technology Centre, Environment Canada 335 River Road, Ottawa, Ontario, 2003). Document: EPA/600/R-03/072.
2. Gros, J. *et al.* First day of an oil spill on the open sea: Early mass transfers of hydrocarbons to air and water. *Environ Sci Technol* **48**, 9400-9411(2014).
